# Supplementary material for: Dickkopf-3 links HSF1 and YAP/TAZ signalling to control aggressive behaviours in cancer-associated fibroblasts
Source: Nat Commun. 2019 Jan 10;10:130. doi: 10.1038/s41467-018-07987-0 (PMC6328607; doi:10.1038/s41467-018-07987-0)
Supplement: Supplementary file 1 — Supplementary Information [file 41467_2018_7987_MOESM1_ESM.pdf]

Dickkopf-3 links HSF1 and YAP/TAZ signalling to control aggressive behaviours in cancer-associated fibroblasts

Nicola Ferrari, Romana Ranftl, Ievgeniia Chicherova, Neil D. Slaven, Emad Moeendarbary, Aaron J. Farrugia, Maxine Lam, Maria Semiannikova, Marie C. W. Westergaard, Julia Tchou, Luca Magnani and Fernando Calvo

SUPPLEMENTARY INFORMATION FILE

This file includes:

- 9 Supplementary Figures
- 6 Supplementary Tables

Ferrari *et al*. Supplementary Information. Supplementary Figure 1.

a

Gene	Tumour	Dataset (GSE)	Probe	Levels in Cancer	p value
DKK3	Breast	Finak (GSE9014)	A_23_P162047	UP	0.00019
	Breast	Karnoub (GSE8977)	214247_s_at	UP	0.00069
	Ovary	Yeoung (GSE40595)	214247_s_at	UP	0.04308
	Colon	Nishida (GSE35602)	A_24_P918317	UP	0.00282
	Aesophagus	Saadi (GSE19632)	10023824931	UP	0.00979
	Oral SCC	Costea (GSE38517)	214247_s_at	UP	0.26958
	Lung	Navab (GSE22863)	3363266	UP	0.5936
	Pancreas	Sherman (GSE43770)	RNA-Seq	UP	0.82795
DKK1	Breast	Finak (GSE9014)	A_23_P24129	DOWN	3.6E-07
	Breast	Karnoub (GSE8977)	204602_at	UP	0.05164
	Ovary	Yeoung (GSE40595)	204602_at	DOWN	0.13177
	Colon	Nishida (GSE35602)	A_23_P24129	UP	0.90474
	Aesophagus	Saadi (GSE19632)	10023818022	UP	0.1711
	Oral SCC	Costea (GSE38517)	204602_at	DOWN	0.5101
	Lung	Navab (GSE22863)	3247172	UP	0.15175
	Pancreas	Sherman (GSE43770)	RNA-Seq	UP	0.06431
DKK2	Breast	Finak (GSE9014)	A_23_P155848	UP	7.1E-10
	Breast	Karnoub (GSE8977)	219908_at	DOWN	0.00275
	Ovary	Yeoung (GSE40595)	219908_at	UP	0.01536
	Colon	Nishida (GSE35602)	A_23_P155848	UP	0.01705
	Aesophagus	Saadi (GSE19632)	10023807552	UP	0.01568
	Oral SCC	Costea (GSE38517)	219908_at	UP	0.64252
	Lung	Navab (GSE22863)	2780907	DOWN	0.10135
	Pancreas	Sherman (GSE43770)	RNA-Seq	DOWN	0.40731
DKK4	Breast	Finak (GSE9014)	A_23_P94275	UP	0.90678
	Breast	Karnoub (GSE8977)	206619_at	UP	0.51042
	Ovary	Yeoung (GSE40595)	206619_at	DOWN	0.07289
	Colon	Nishida (GSE35602)	A_23_P94275	DOWN	0.0131
	Aesophagus	Saadi (GSE19632)	10025904682	DOWN	0.93149
	Oral SCC	Costea (GSE38517)	206619_at	UP	0.22534
	Lung	Navab (GSE22863)	3133325	UP	0.01657
	Pancreas	Sherman (GSE43770)	RNAseq	UP	nd
DKKL1	Breast	Finak (GSE9014)	A_23_P130743	UP	9E-15
	Breast	Karnoub (GSE8977)	220284_at	DOWN	0.2342
	Ovary	Yeoung (GSE40595)	220284_at	DOWN	0.05332
	Colon	Nishida (GSE35602)	A_23_P130743	DOWN	0.00539
	Aesophagus	Saadi (GSE19632)	10025912532	UP	0.62779
	Oral SCC	Costea (GSE38517)	220284_at	DOWN	0.27465
	Lung	Navab (GSE22863)	3838425	UP	0.02686
	Pancreas	Sherman (GSE43770)	RNAseq	DOWN	0.77041

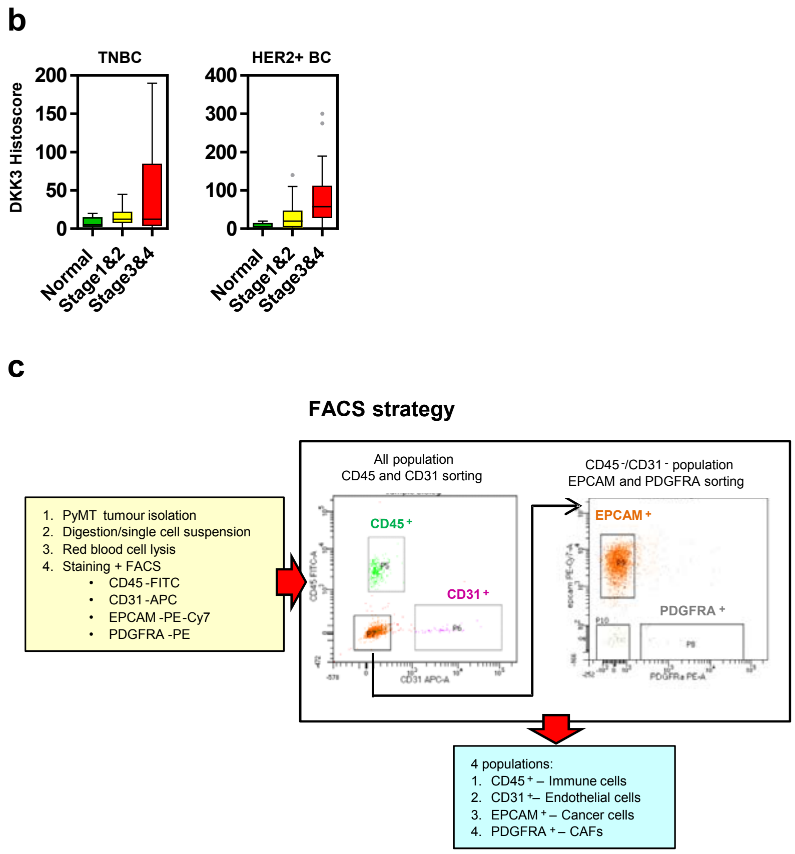

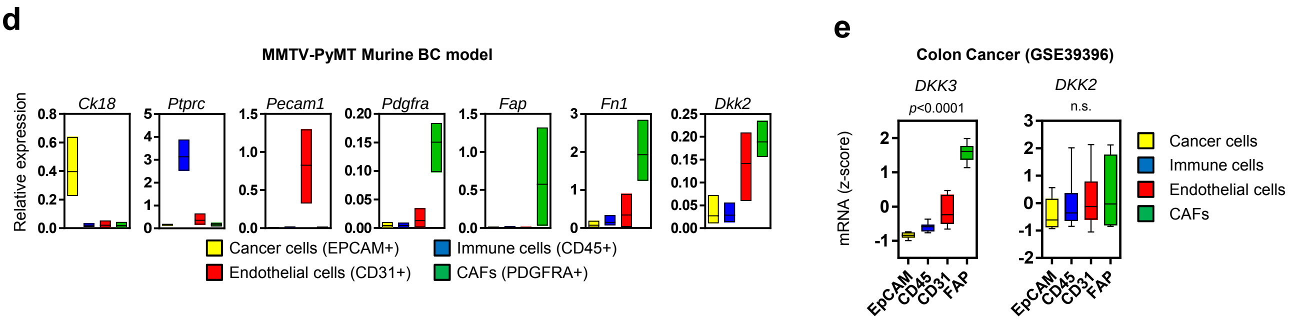

Supplementary Figure 1. DKK3 expression in the tumour stroma. (a) Table showing the differential levels of expression of Dickkopft genes (*DKK3*, *DKK1*, *DKK2*, *DKK4* and *DKKL1*) between normal and cancerous stroma in different types of tumours (breast, ovary, colon, aesophagus, oral squamous cell carcinoma, lung and pancreas). Public datasets used to extract the information and the specific probe are indicated. Additional information includes whether genes are upregulated/downregulated in cancer stroma; colours range from dark green (significantly upregulated), light green (non-significantly upregulated), light red (non-significantly downregulated) and dark red (significantly upregulated). *p* values for each probe and dataset are provided; colours range from orange (highly significant), yellow (significant) to white (non-significant). **(b)** Tukey boxplots showing quantification of DKK3 staining (Histoscore) in non-invasive breast cancers (Stage 1&2), invasive breast-cancers (Stage 3&4) and normal tissue counterparts. Left graph shows triple-negative breast cancers (TNBC) and right graph shows HER2-positive breast cancers (TNBC: normal, *n*=9; Stage 1&2, *n*=10; Stage 3&4, *n*=10. HER2-positive: normal, *n*=9; Stage 1&2, *n*=30; Stage 3&4, *n*=30). **(c)** Diagram showing the FACS gating strategy to isolate different cell populations from MMTV-PyMT murine mammary tumours. **(d)** Expression of indicated genes (relative to *Gapdh*) in different cell populations isolated from MMTV-PyMT mammary tumours: Cancer cells (Epcam$^+$), immune cells (Cd45$^+$), endothelial cells (Cd31$^+$) and fibroblasts (Pdgfra$^+$). Transcripts for the cancer cell marker Ck18, immune cell marker Ptprc, endothelial cell marker Pecam1 and fibroblast markers Pdgfra, Fap and Fn1 were enriched in their corresponding cell populations, demonstrating successful cell fractionation. Expression of Dickkopf gene Dkk2 is also shown. Expression of *Dkk2* is also shown. Floating boxes: centre line, mean; box limits, min and max values (n=4 individual tumours for all graphs, except *Ck18*, *n*=3; and *Pdgfra*, *Fap* and *Fn1*, *n*=5). **(e)** Tukey boxplot showing *DKK3* and *DKK2* mRNA expression levels (z-score) in FACS sorted cell populations isolated from human colorectal tumours: Cancer cells (Epcam$^+$), immune cells (CD45$^+$), endothelial cells (CD31$^+$) and fibroblasts (FAP+). From GSE39396; (*n*=6 individual tumours).

Ferrari *et al*. Supplementary Information. Supplementary Figure 2.

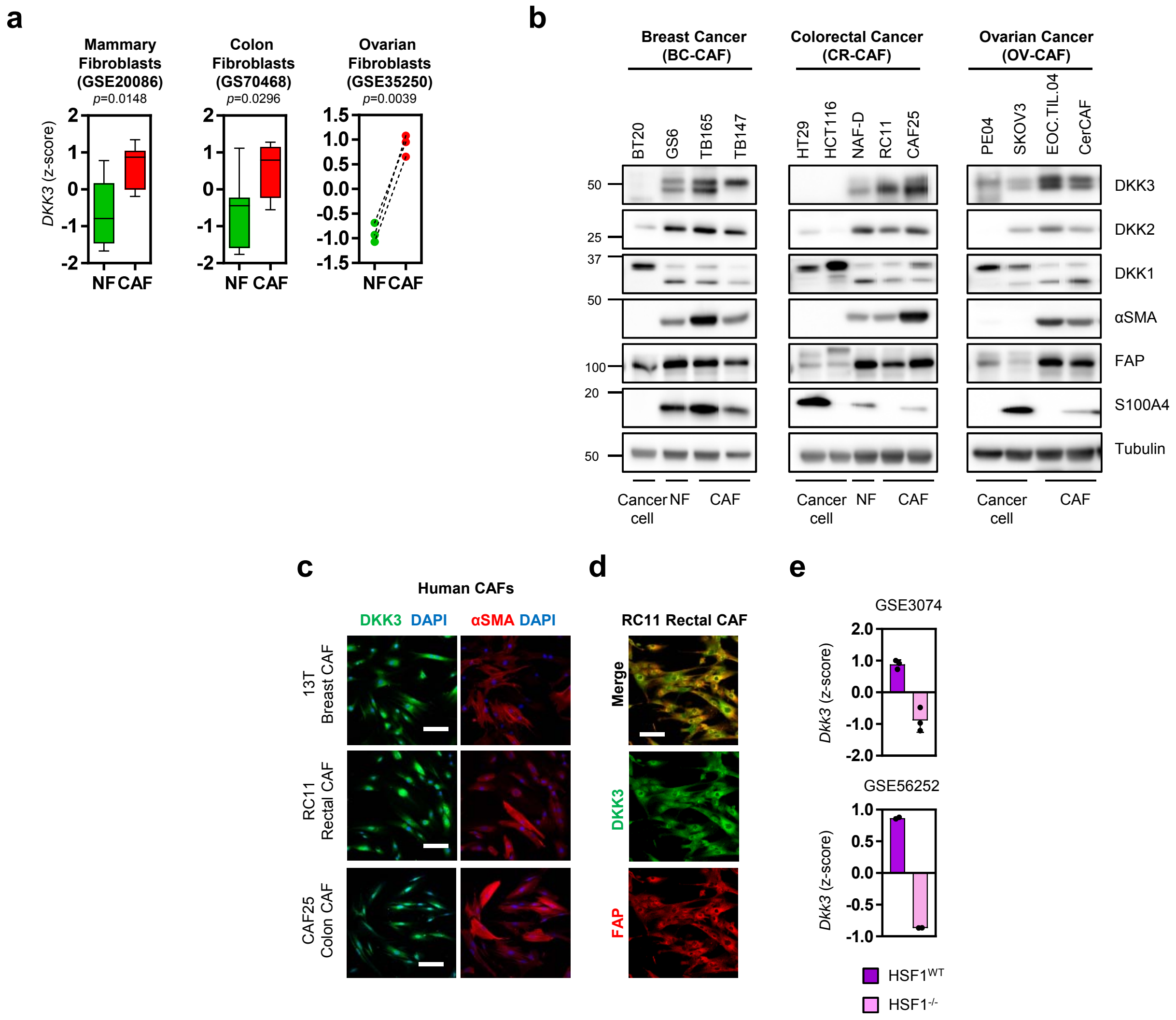

Supplementary Figure 2. DKK3 expression in human CAFs. (a) (Left and middle) Tukey boxplots showing z-score values of *DKK3* mRNA expression in NFs and CAFs from human breast (*left,* extracted from GSE20086; *n*=6) and human colon (*middle*, extracted from GSE70468; *n*=7). Right graph shows z-score values of *DKK3* mRNA expression in matched human ovarian NFs and CAFs (extracted from GSE35250, *n*=3). *p* values are shown. **(b)** Western blot showing levels of DKK3, DKK2, DKK1, αSMA, FAP, S100A4 and tubulin in total lysates of human breast, colon and ovarian cancer NFs and CAFs. Where indicated, expression levels of cancer-type-matched cancer lines are also shown. **(c)** (Left panels) Images show DKK3 (green) and DAPI (blue) staining of human breast CAFs (13T), rectal CAFs (RC11) and colon CAFs (CAF25). (Right panels) Images show same cells counterstained for αSMA (red) and DAPI (blue). Scale bars, 150 µm. **(d)** Images show DKK3 (green) and FAP (red) staining of human colon CAFs (RC11). Upper panel shows merged image. Scale bar, 150 µm. **(e)** Charts show *Dkk3* mRNA expression (shown as z-score) in wild-type (HSF1^{wt}) and *Hsf1*-null (HSF1$^{-/-}$) MEFs in two independent datasets (GSE3074 and GSE56252). Bars represent mean value ± SD.

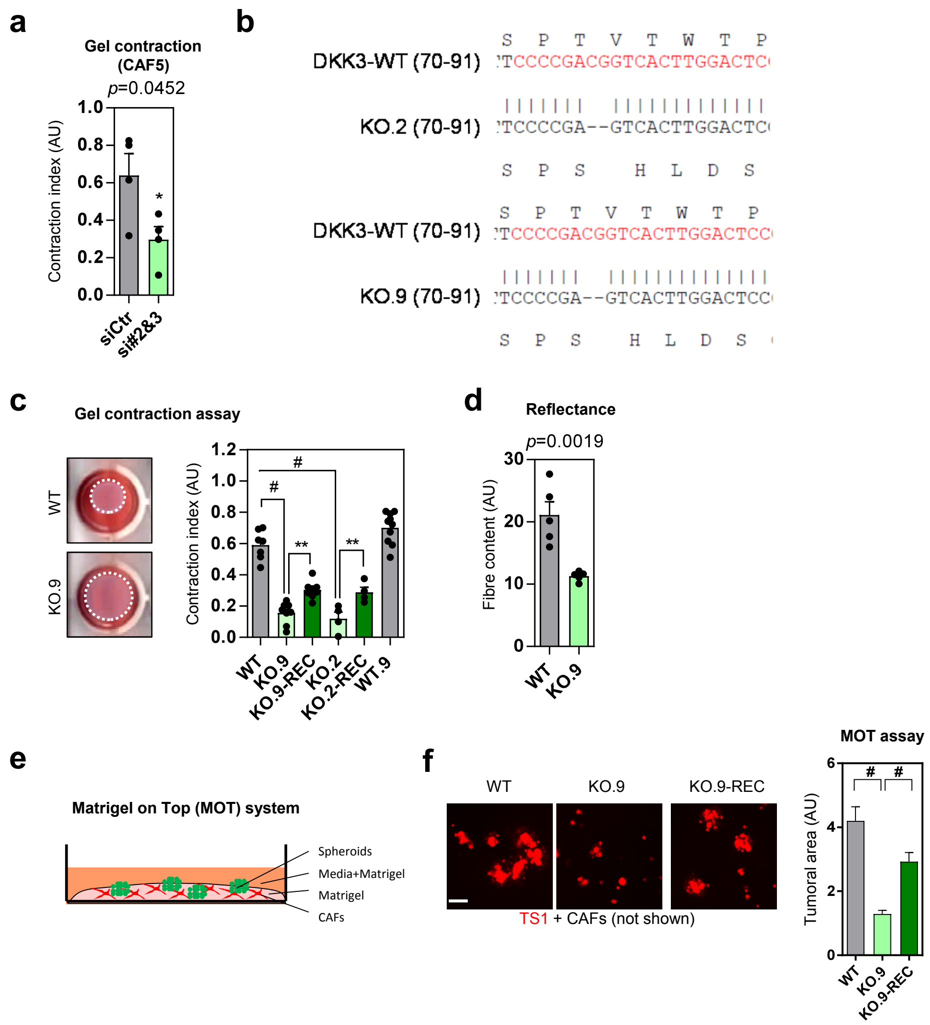

Supplementary Figure 3. DKK3 modulates CAF functions. (a) Graph shows gel contraction by CAF5 after transfection with control (siCtr) and DKK3 (si#2&3) siRNAs. Bars represent mean ± SEM (n=5 individual gels). **(b)** Diagram showing the targeting sequence (red) for endogenous *Dkk3* CRISPR/CAS9 knock-out in murine wild-type CAFs. Underneath, sequences of the same *Dkk3* locus on KO.9 and KO.2 CAFs, showing the targeted deletion of two single bases. **(c)** Images show gels remodelled by WT and KO.9 CAFs, with a dashed white line delineating the final gel area. Histogram shows gel contraction by the indicated CAF lines. Gel remodelling activity of a CAF1 DKK3-positive clone (WT.9) is also shown. Bars represent mean ± SEM (6<n<10 individual gels). **(d)** Histogram shows fibre content (i.e. reflectance intensity) in gels remodelled by WT and KO.9 CAFs. Bars represent mean ± SEM (n=5). **(e)** Cartoon describing the experimental set-up for the *Matrigel on top* (MOT) co-culture system. Cancer cells (green) alone or mixed with fibroblasts (red) are seeded on top of a thin layer of Matrigel (pink) and fed with media containing 10% FBS and 2% Matrigel (orange). **(f)** Representative images of end-point TS1 murine breast cancer cells (red) obtained after MOT co-culture with WT, KO.9 and KO.9-REC CAFs (not shown). Scale bar, 250 µm. Graph shows the *tumoral area* as measured from the size of colonies. Bars represent mean ± SEM (*n*>28 individual colonies out of 3 independent experiments).

Ferrari *et al*. Supplementary Information. Supplementary Figure 4.

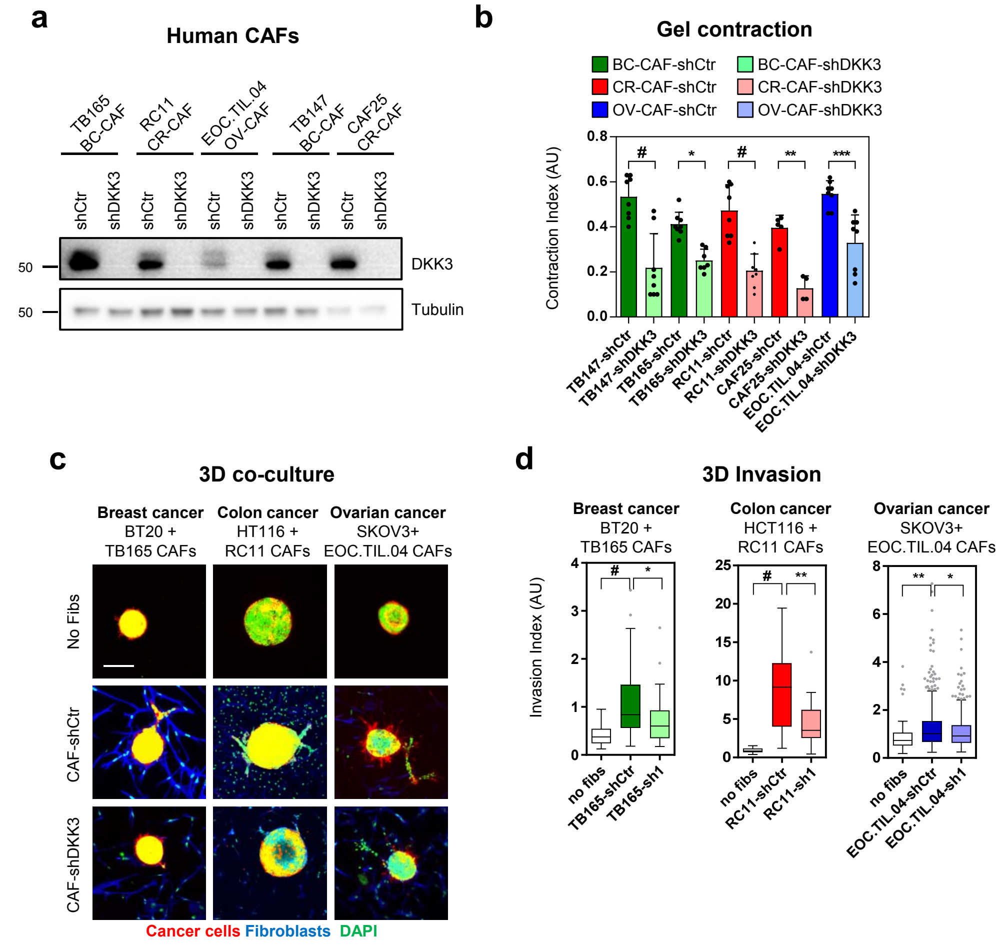

Supplementary Figure 4. DKK3 promotes pro-tumorigenic functions in human CAFs. (a) Western blot showing levels of DKK3 and tubulin in human breast CAFs (TB165-BC-CAFs and TB147-BC-CAFs), colorectal CAFs (RC11-CR-CAFs and CAF25-CR-CAF) and ovarian CAFs (EOC.TIL.04-OV-CAFs) stably expressing control (shCtr) and DKK3 (shDKK3) shRNAs. **(b)** Histogram showing gel contraction by the indicated human CAF lines (breast, green bars; colorectal, red bars; ovarian, blue bars) stably expressing control (dark coloured bars) or DKK3 (light coloured bars) shRNAs. Bars represent mean ± SEM (n=4 or more individual gels out of at least 3 independent experiments). **(c)** Images show representative end-point spheroids of breast cancer (BT20), colon cancer (HCT116) and ovarian cancer (SKOV3) cells (red) obtained after 3D co-culture with tumour-matched CAFs (TB165, RC11 and EOC.TIL.04, respectively) stably expressing control or DKK3 shRNA (blue). Spheroids obtained by mono-culture (i.e. no fibroblasts added) are also shown. DAPI staining (green) was also used. Scale bar, 200 μm. **(d)** Tukey boxplots show the *invasion index* (3D invasion) measured from spheroids described in (c); n=15 or more spheroids out of at least 3 independent experiments.

Ferrari *et al*. Supplementary Information. Supplementary Figure 5.

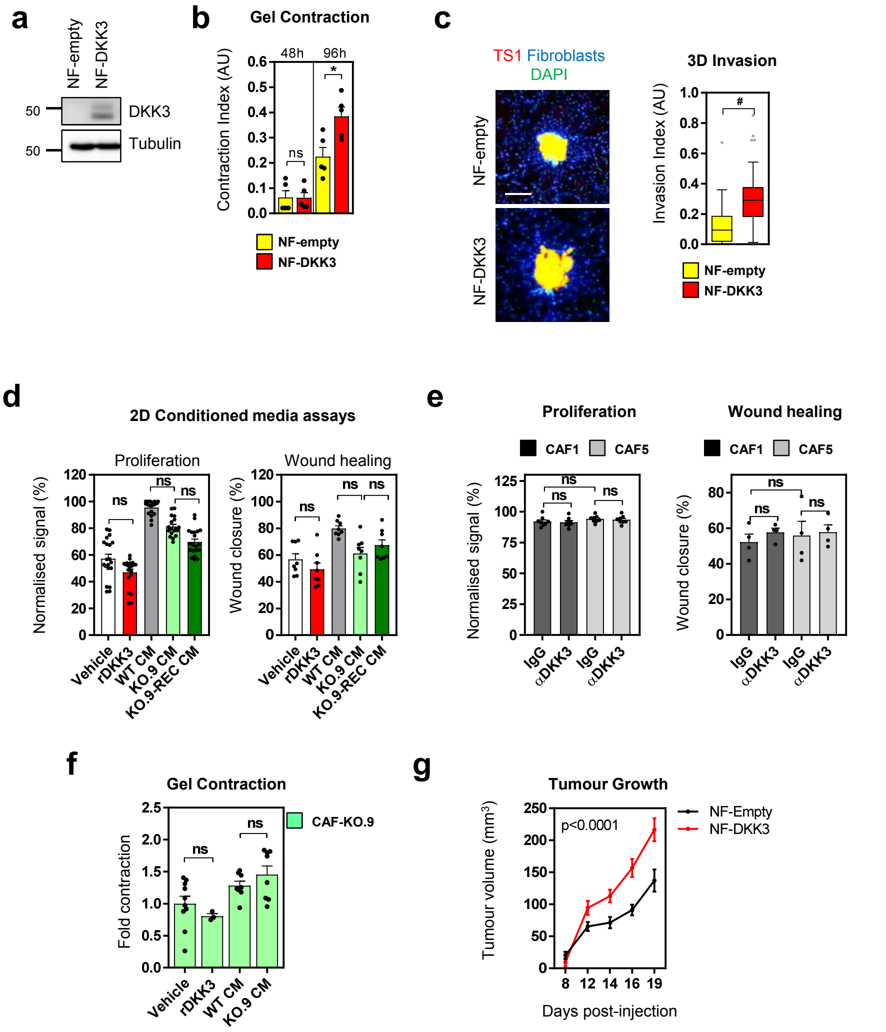

Supplementary Figure 5. Ectopic expression of DKK3 in NFs induces an activated phenotype. (a) Western blot showing levels of DKK3 and Tubulin in murine NF after stable ectopic expression of DKK3 or empty vector. **(b)** Graph showing gel contraction at 48 and 96 h of NF-empty and NF-DKK3. Bars represent mean ± SEM (n=5 individual gels, 2 individual experiments). **(c)** Images show representative end-point TS1 spheroids (red) after 3D co-culture with NF-empty and NF-DKK3 (blue). DAPI staining (green) was also used. Scale bar, 200 µm. Tukey boxplot shows the *invasion index; n*>25 individual spheroids out of 3 independent experiments. **(d)** Histograms showing proliferation index (left) and migration in wound healing assays (right) of TS1 cells when cultured in normal media (vehicle), media containing 100 ng mL^{-1} recombinant DKK3 (rDKK3) or conditioned media (CM) obtained from WT, KO.9 and KO.9-REC CAFs. Bars represent mean ± SEM (proliferation: n=16 replicates, 4 independent repeats; migration, n=8 replicates, 4 independent repeats). **(e)** Histograms showing proliferation index (left) and migration in wound healing assays (right) of TS1 cells when cultured in conditioned media from CAF1 (with detectable secreted DKK3) or CAF5 (no detectable secreted DKK3, *See Figure 2b*). As indicated, cells were cultured in the presence of 1 µg mL^{-1} of a blocking antibody against DKK3 (αDKK3) or with isotype control antibody (IgG). Bars represent mean ± SEM (proliferation: n=8 replicates, 4 independent repeats; migration: n=4 independent repeats). **(f)** Effect of recombinant DKK3 and DKK3-containing medium on CAF-KO.9. Graph shows the gel contraction index of CAF-KO.9 when cultured in normal media (vehicle), media containing 100 ng mL^{-1} recombinant DKK3 (rDKK3) or conditioned media (CM) obtained from WT or KO.9 CAFs. Bars represent mean ± SEM (3<n<10). **(g)** Graph showing the volumes of tumours generated by co-injection of TS1 cells with NF-empty or NF-DKK3 in syngeneic mice (FVB/n) at the indicated days post-injection. Lines represent mean ± SEM (*n*=8 for NF-empty; *n*=7 for NF-DKK3). For all graphs, *, *P* < 0.05; **, *P* < 0.01; ***, *P* < 0.001; #, *P* < 0.0001; n.s., non-significant.

Ferrari *et al*. Supplementary Information. Supplementary Figure 6.

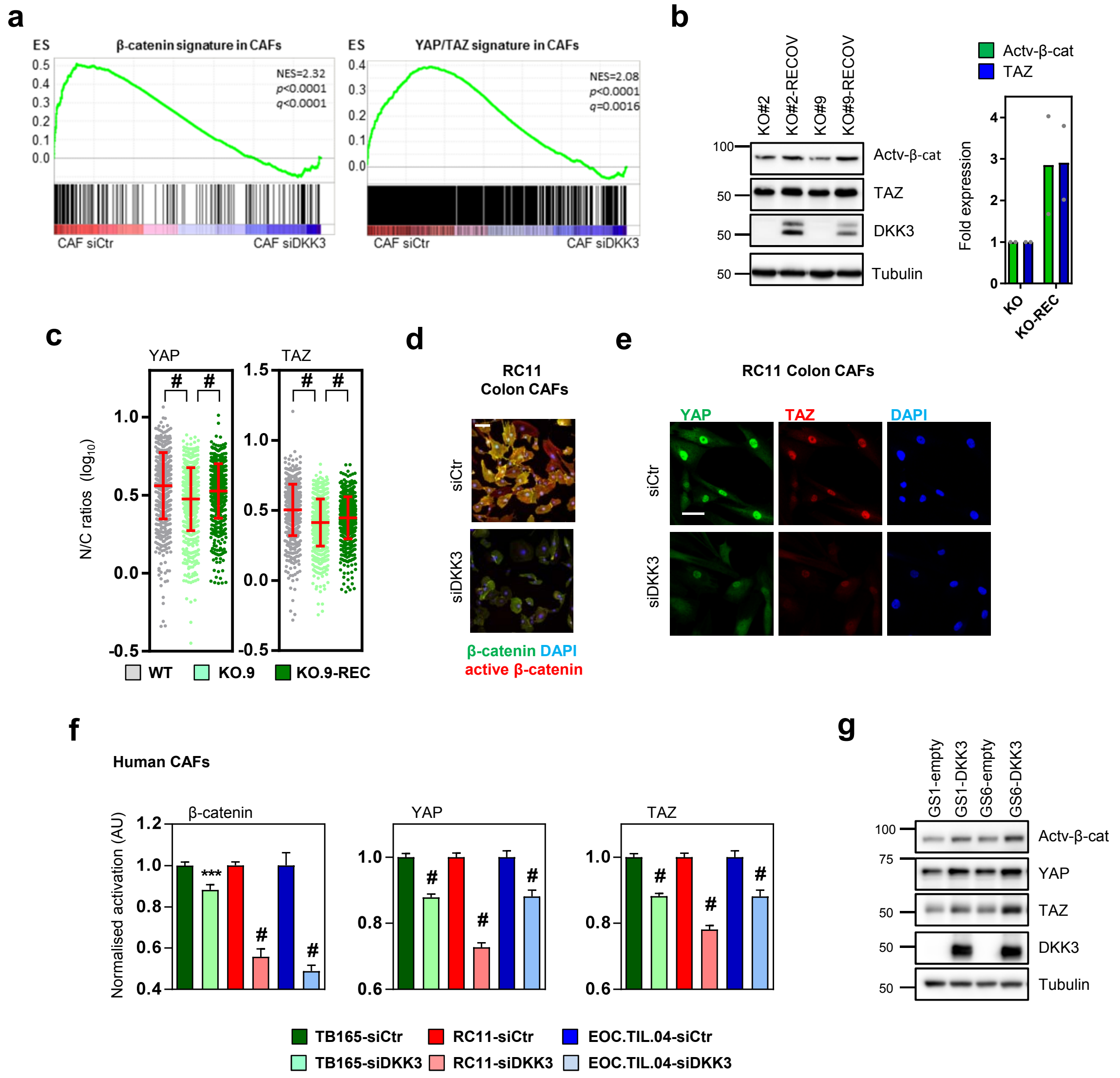

Supplementary Figure 6. DKK3 activates β-catenin and YAP/TAZ in CAFs. (a) GSEA plots show the enrichment of β-catenin and YAP/TAZ target genes in CAF-siCtr vs CAF-siDKK3. Nominal Enrichment Score (NES), False discovery rate (FDR) *q*-value and standard *p*-value are shown for both plots. **(b)** Western blot showing non-phospho (active) β-catenin (Ser33/37/Thr41), TAZ, DKK3 and Tubulin levels in KO.2, KO.2-REC, KO.9, KO.9-REC CAFs. Graph represents quantification of the blots showing the amount of active-β-catenin and TAZ (normalised to tubulin) in KO-REC relative to KO CAFs (*n*=2). **(c)** Graphs show quantification of nuclear relative to cytosolic fluorescent intensity (log_{10} ratios) of YAP or TAZ in murine WT, KO.9 and KO.9-REC CAFs Each dot represents a single cell from 3 independent experiments. Lines represent mean ± SEM. **(d)** Images show total β-catenin (green), active-β-catenin (red), and DAPI (blue) staining of human colorectal RC11 CAFs after transfection with control (siCtr) or DKK3 siRNA (smart-pool). Scale bar, 100 µm. **(e)** Images shown YAP (green) and TAZ (red) localization and DAPI staining (blue) in human colorectal RC11 CAFs after transfection with control (siCtr) or DKK3 siRNA (smart-pool). Scale bars, 50 µm. **(f)** Graphs show normalized β-catenin, YAP and TAZ activation in human CAFs (TB165, breast cancer, green bars; RC11, rectal, red bars; EOC.TIL.04, ovarian, blue bars) after transfection with control (siCtr, dark colour) or DKK3 siRNA (smart-pool; siDKK3, light colour). Bars represent mean ± SEM (*n*=19 or more fields of view for β-catenin; *n*=298 or more single cells for YAP and TAZ analysis). **(g)** Representative Western blots showing non-phospho (active) β-catenin (Ser33/37/Thr41), YAP, TAZ, DKK3 and Tubulin in human mammary NFs (GS1 and GS6) after stable ectopic expression of empty vector or DKK3.

Ferrari *et al*. Supplementary Information. Supplementary Figure 7.

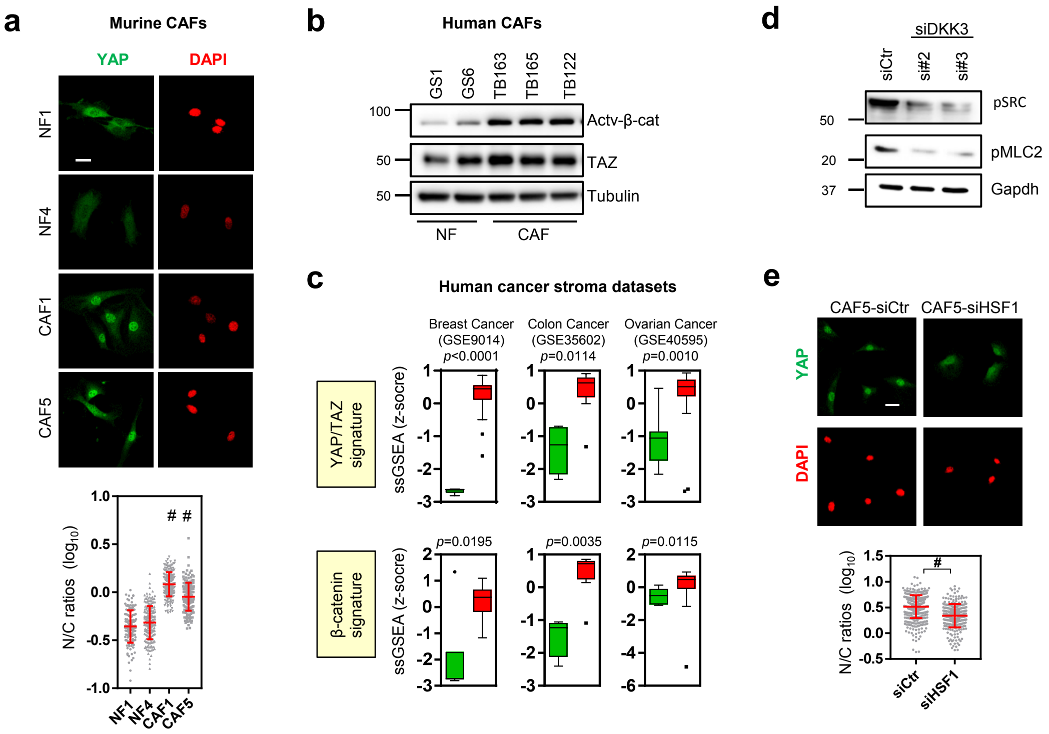

Supplementary Figure 7. CAFs present constitutive activation of β-catenin and YAP/TAZ signalling. (a) Images shown YAP localization (left panels, green) and DAPI staining (right panels, red) in murine NFs (NF1 and NF4) and PyMT-CAFs (CAF1 and CAF5). Scale bar, 70 µm. Bottom graph shows quantification of nuclear relative to cytosolic fluorescent intensity (log_{10} ratios) of YAP. Each dot represents a single cell from 3 independent experiments. Lines represent mean ± SEM. **(b)** Representative Western blot showing non-phospho (active) β-catenin (Ser33/37/Thr41), TAZ and Tubulin in human mammary NFs (GS1 and GS6) and CAFs (TB163, TB165, TB122). **(c)** Tukey boxplots showing z-score values of YAP/TAZ and β-catenin CAF-specific gene signatures in normal and cancerous stroma from breast (GSE9014), colorectal (GSE35602) and ovarian (GSE40595) cancers. Individual *p* values are shown (Breast: normal, *n*=6; cancer, *n*=53. Colon: normal, *n*=4; cancer, *n*=13. Ovary: normal, *n*=8; cancer, *n*=31). **(d)** Western blot showing pY416-Src (pSRC), pS19-MLC2 (pMLC2) and Gapdh in CAF1 following transfection with control (siCtr) and two different DKK3 siRNAs (si#2 and si#3). **(e)** Images shown YAP localization (upper panels, green) and DAPI staining (lower panels, red) in murine PYMT-CAF5 after transfection with control (siCtr) or Hsf1 (smart-pool) siRNAs. Scale bar, 70 µm. Bottom graph shows quantification of nuclear relative to cytosolic fluorescent intensity (log_{10} ratios) of YAP. Each dot represents a single cell from 3 independent experiments. Lines represent mean ± SEM. For all graphs, *, *P* < 0.05; **, *P* < 0.01; ***, *P* < 0.001; #, *P* < 0.0001; n.s., non-significant

Ferrari *et al*. Supplementary Information. Supplementary Figure 8.

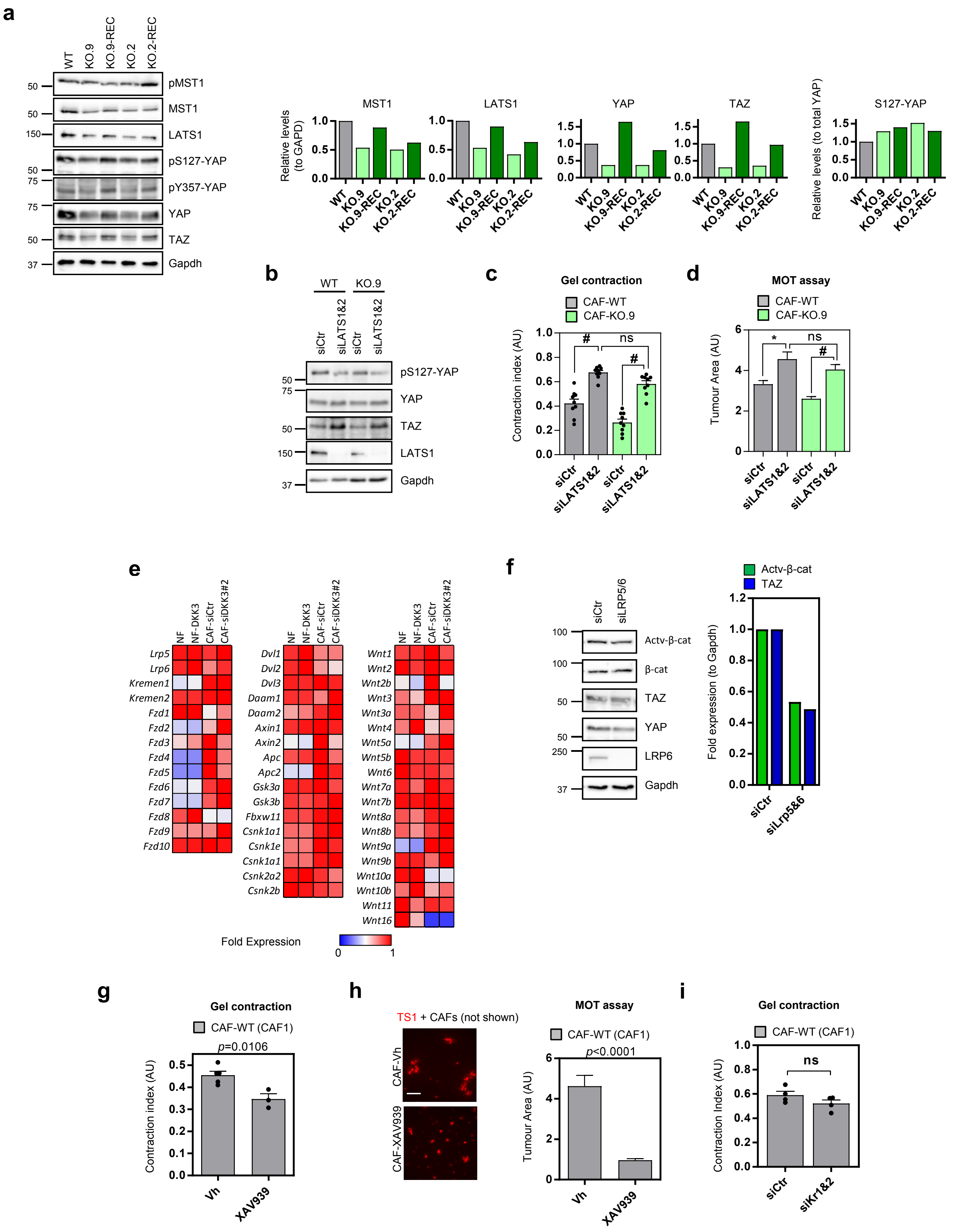

Supplementary Figure 8. DKK3 regulation of YAP/TAZ is independent of the Hippo pathway. (a) Western blot showing levels of pT183/181-MST1/2, MST1, MST2, LATS, pS127-YAP, pY357-YAP, YAZ, TAZ and Gapdh in wild-type CAF1 (WT) and two sets of DKK3 knock-out CAF1 clones (KO.9 and KO.2) and their recovery counterparts where DKK3 was stably re-expressed (KO.9-REC and KO.2-REC). Graphs on the right represent quantification of the blots showing the relative amount of MST1, LATS1, YAP, TAZ normalised to GAPDH, and pS127-YAP levels normalised to total YAP. **(b**) Western blot showing levels of pS127-YAP, YAP, TAZ, LATS1 and Gapdh in wild-type CAF1 (WT) and KO.9 CAFs after transfection with control (siCtr) or Lats1&2 (smart-pool) siRNAs. **(c)** Graph shows gel contraction index of murine wild-type (WT) and KO.9 CAFs after transfection with control (siCtr) or Lats1&2 (smart-pool) siRNAs. Bars represent mean ± SEM (n=9 individual gels from 3 independent experiments). **(d)** Graph shows the "tumoral area" of TS1 murine breast cancer cells obtained after MOT co-culture with wild-type CAF1 (WT) or KO.9 CAFs after transfection with control (siCtr) or Lats1&2 (smart-pool) siRNAs. Bars represent mean ± SEM (n=346 or more colonies from at least 2 independent experiments). **(e)** Colour-coded grid showing the fold expression of genes associated to Wnt signalling in NF, NFs overexpressing DKK3 (NF-DKK3) and CAF1 after transfection with control (siCtr) and DKK3 siRNA. Colours range from red to blue representing respectively the lowest (zero) and highest fold activity (one). Genes are grouped into receptors (left), ligands (right) and core regulators (middle). Data is from microarray analysis, mean value of two independent biological replicates is shown. **(f)** Western blot showing levels of non-phospho (active) β-catenin (Ser33/37/Thr41), β-catenin, YAP, TAZ, LRP6 and Gapdh in CAF1 after transfection with control (siCtr) and LRP5&6 siRNA (smart-pool). Graph represents quantification of blots indicating the fold amount of non-phospho (active) β-catenin (Ser33/37/Thr41) and TAZ (normalised to Gapdh) in CAF1 after transfection with control (siCtr) and LRP5&6 siRNA (smart-pool). **(g)** Graph shows gel contraction index of CAF1 after treatment with vehicle or 2 µM XAV939. Bars represent mean ± SEM (n=3 or more). **(h)** Representative images of end-point TS1 murine breast cancer cells (red) obtained after MOT co-culture with CAF1 previously treated with vehicle (Vh) or 2 µM XAV939 for 48 h. CAFs are not shown. Scale bar, 200 µm. Graph shows the mean area of colonies formed by TS1. Bars represent mean ± SEM (n=24 or more colonies). **(i)** Graph shows gel contraction index of murine WT-CAFs after transfection with control (siCtr) or Kremen1&2 (siKr1&2; smart-pool) siRNAs. Bars represent mean ± SEM (n=4 or more individual gels).

Ferrari *et al*. Supplementary Information. Supplementary Figure 9.

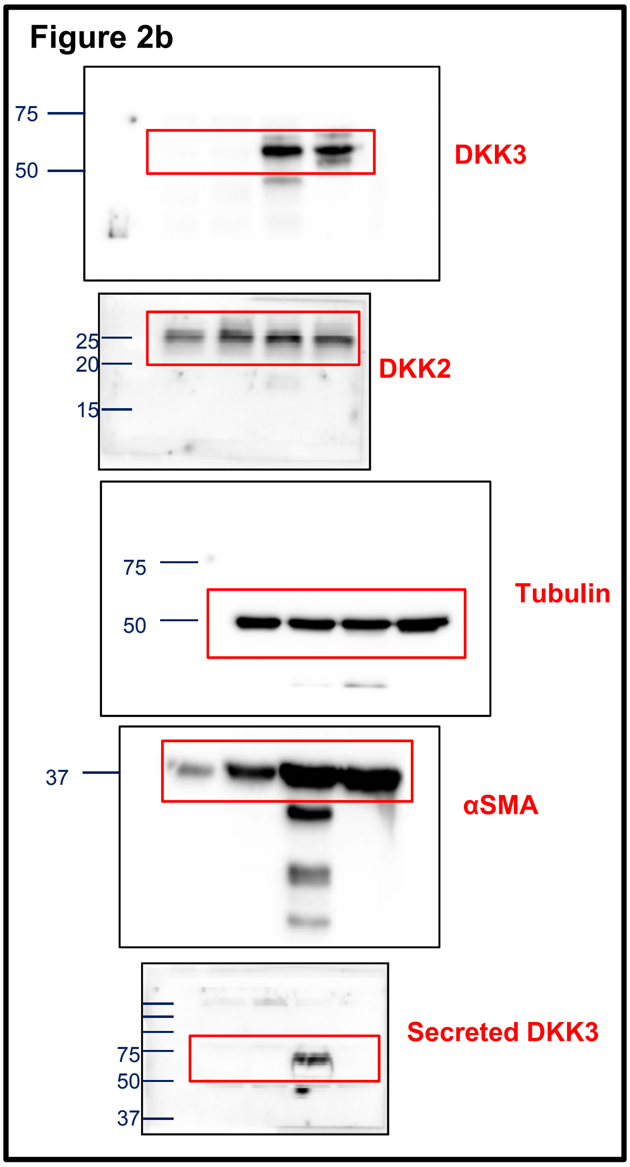

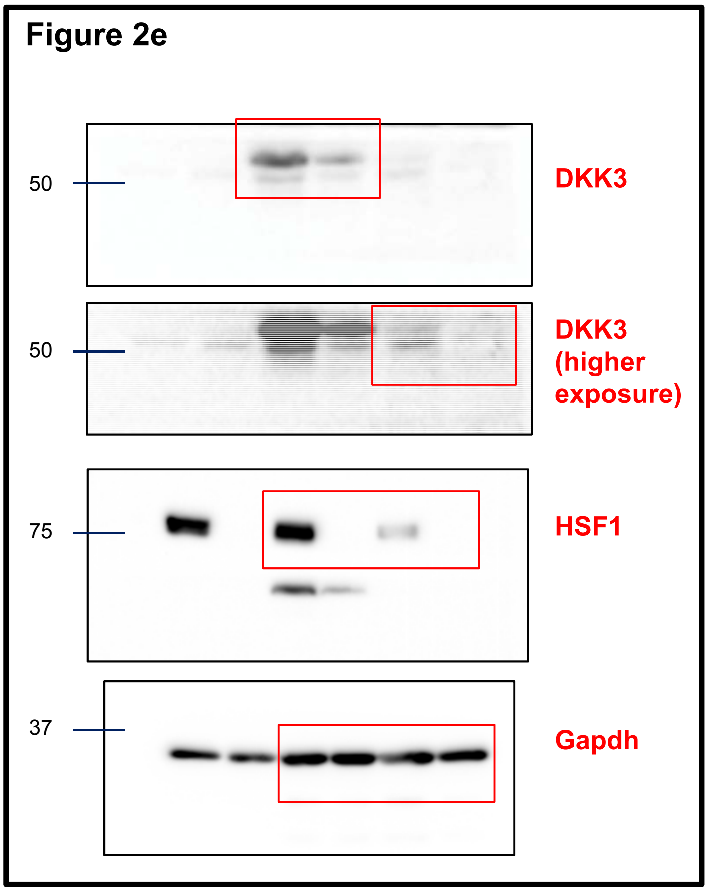

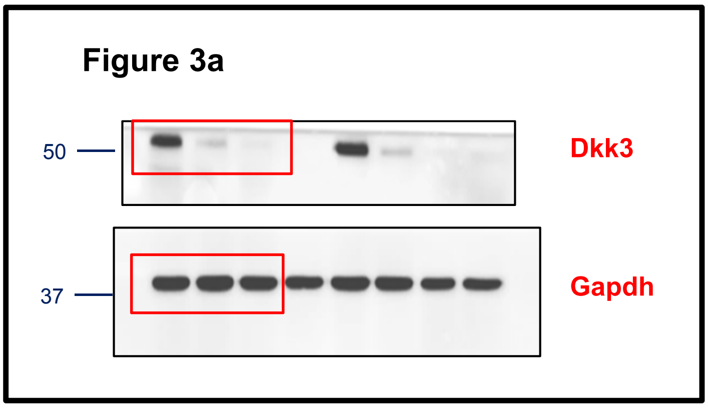

Ferrari *et al*. Supplementary Information. Supplementary Figure 9 (*continued*)

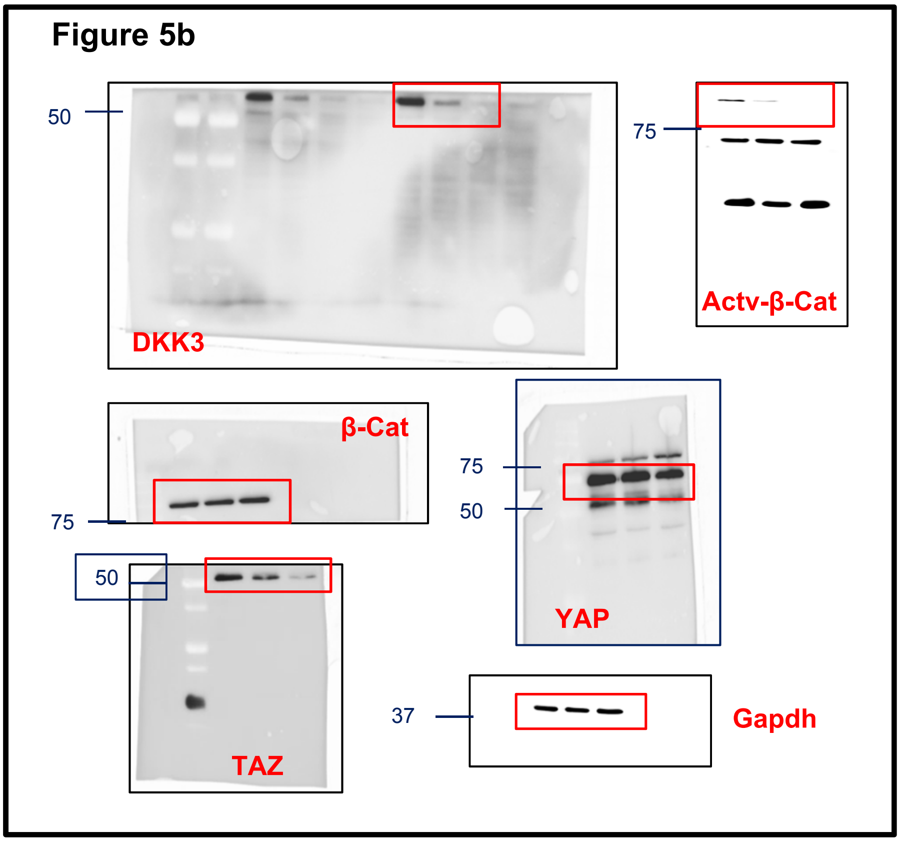

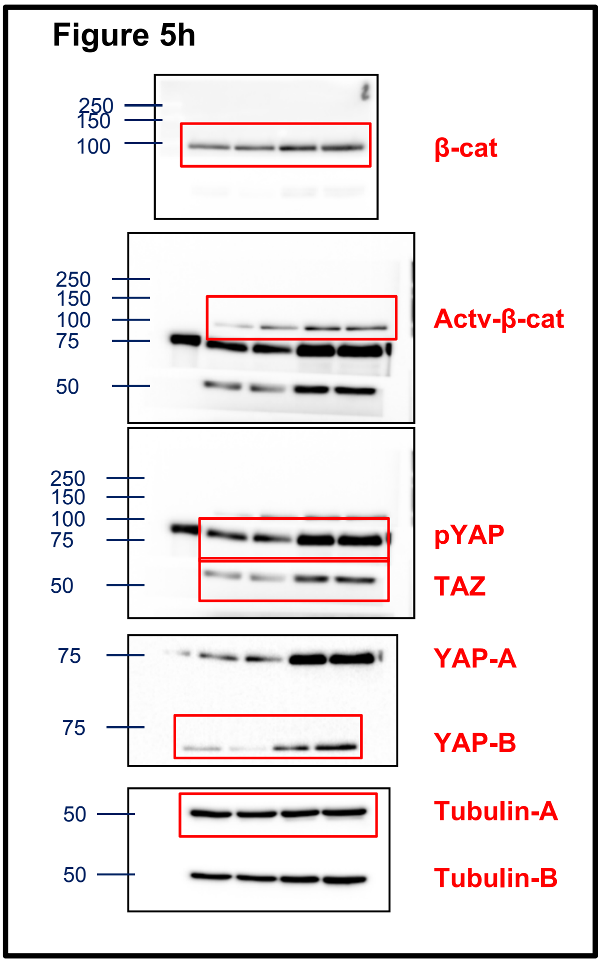

Ferrari *et al*. Supplementary Information. Supplementary Figure 9 (*continued*)

Figure 6a

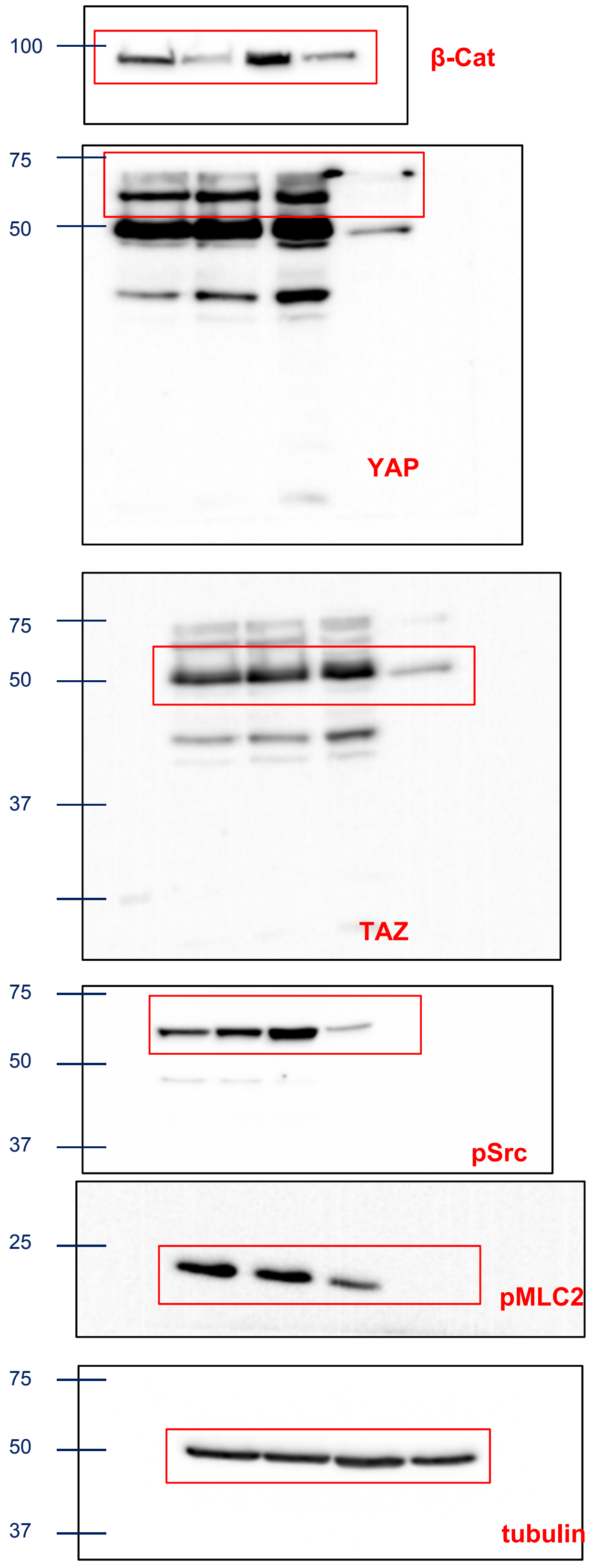

Figure 6i

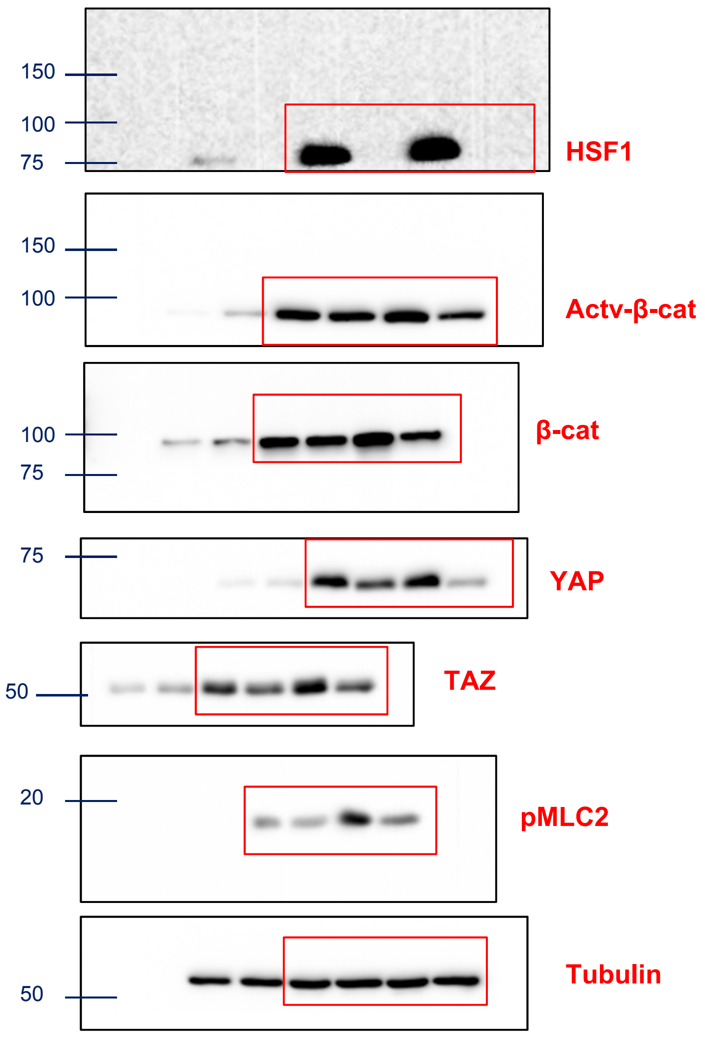

Ferrari *et al*. Supplementary Information. Supplementary Figure 9 (*continued*)

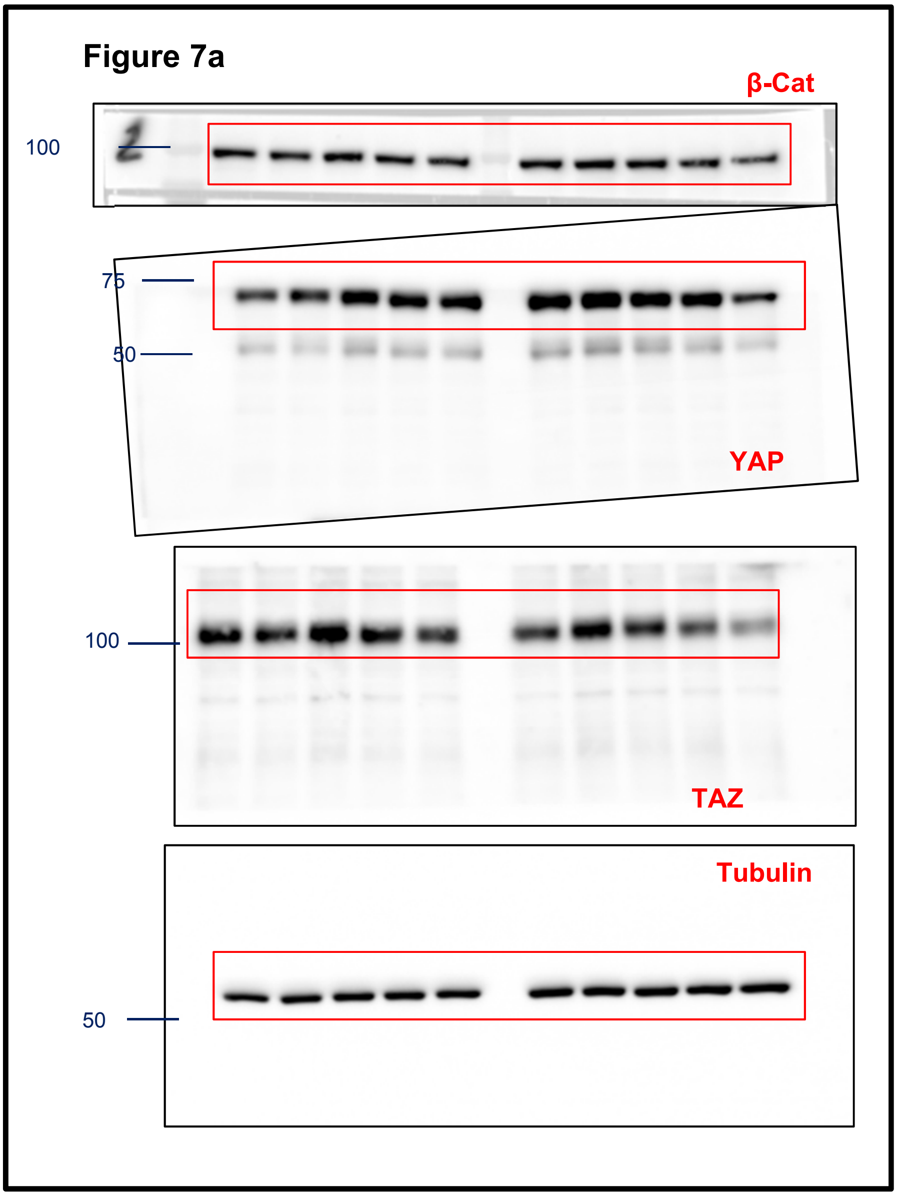

Ferrari *et al*. Supplementary Information. Supplementary Figure 9 (*continued*)

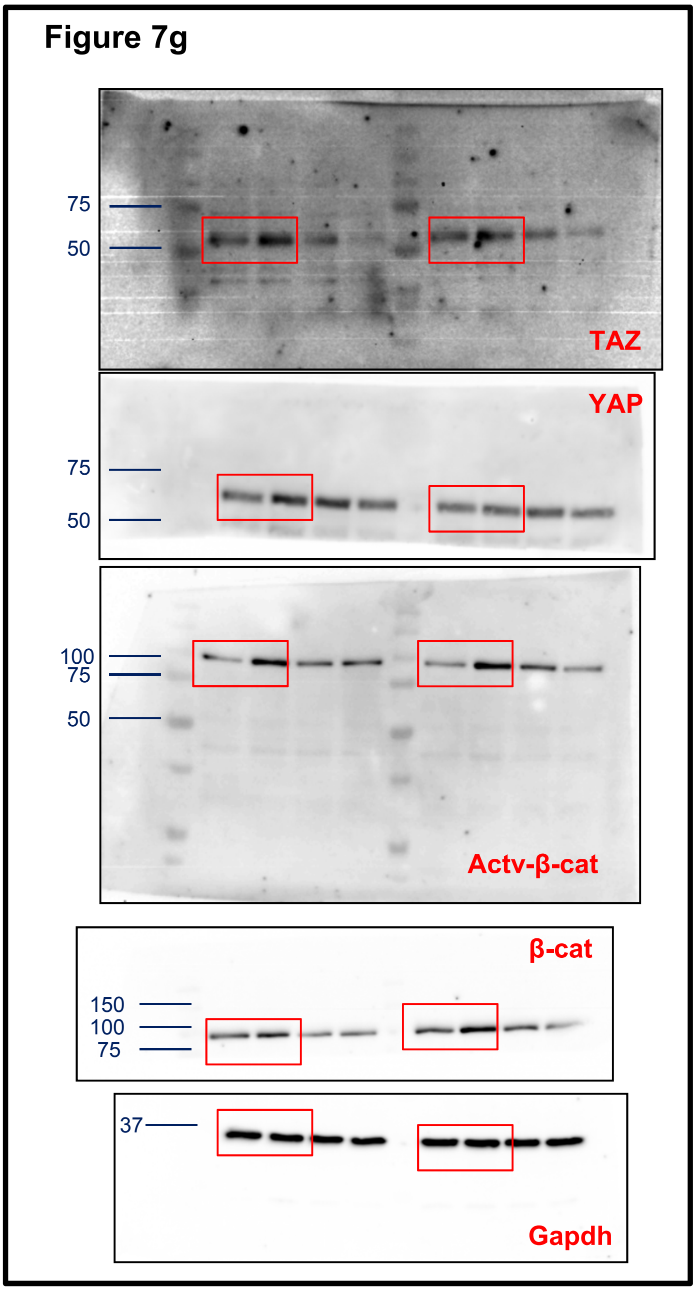

Ferrari *et al*. Supplementary Information. Supplementary Figure 9 (*continued*)

Figure 8a

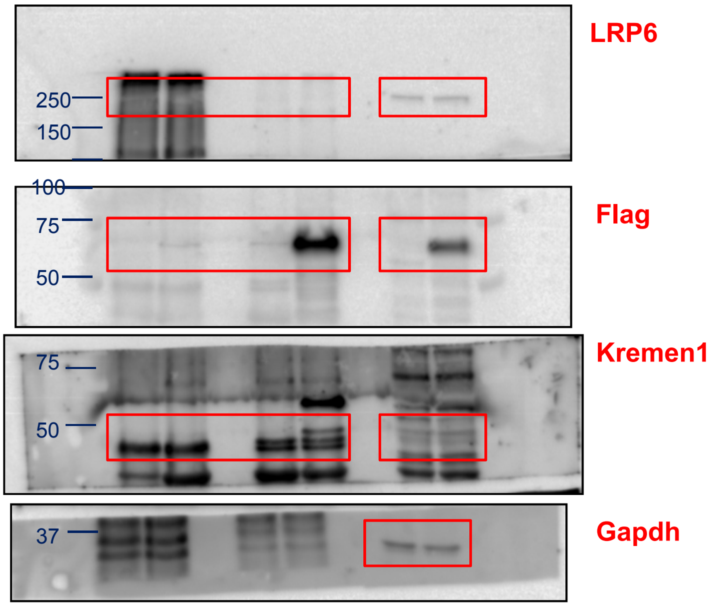

Figure 8d

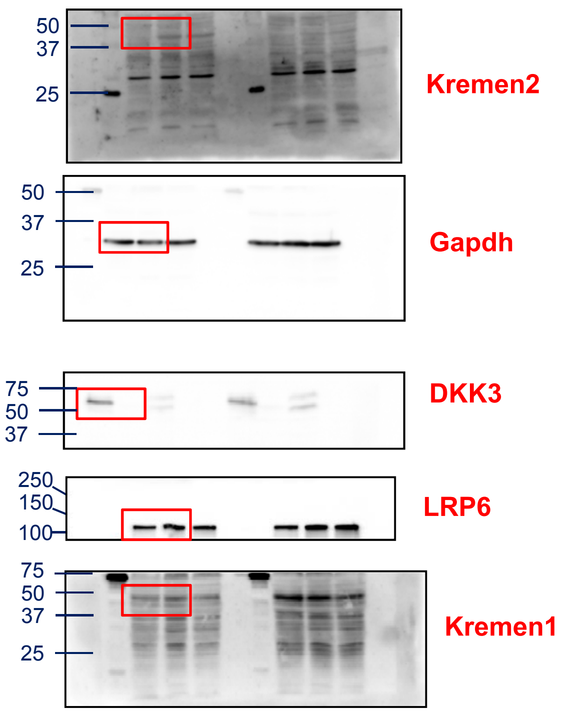

Ferrari *et al*. Supplementary Information. Supplementary Figure 9 (*continued*)

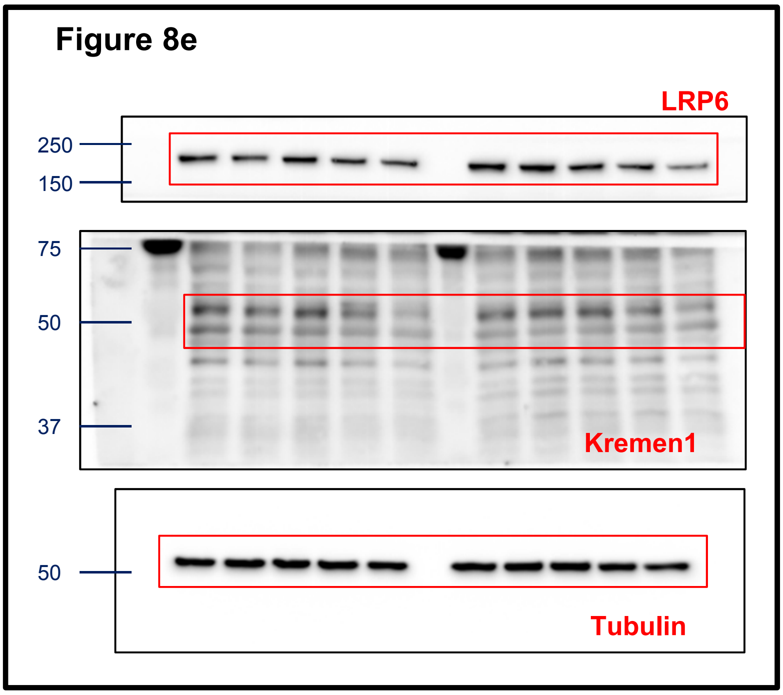

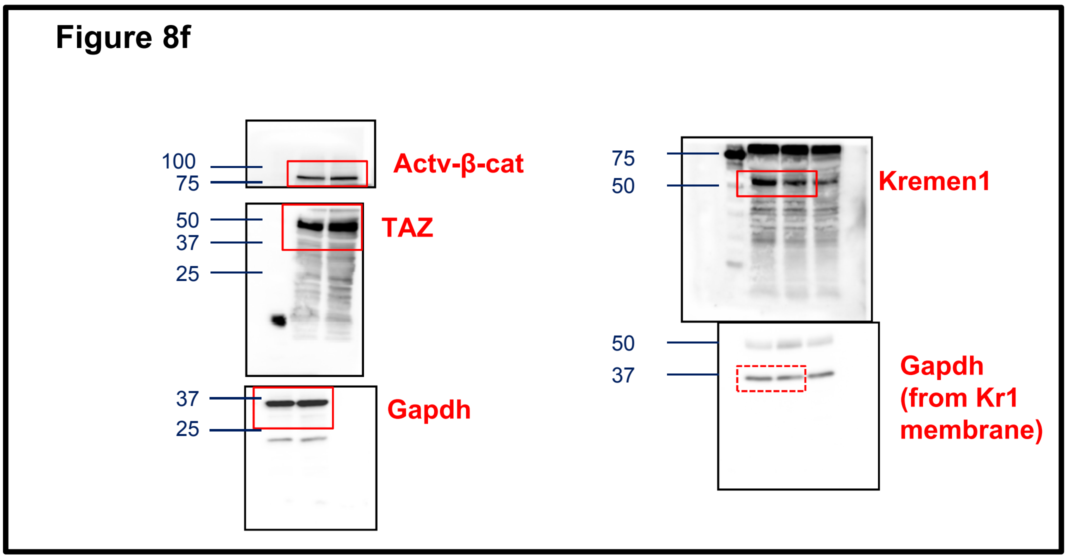

Supplementary Figure 9. Uncropped immunoblot images. Uncropped immunoblot images corresponding to the indicated blots from main Figures. Cropped images are delineated by a red box. Molecular size markers are in kDa.

Ferrari *et al*. Supplementary Information. Supplementary Table 1.

NAME	SIZE	ES	NES	NOM p-val	FDR q-val	LEADING EDGE
CAF_BCAT_ALL_UP	171	0.5100316	2.3684409	0	0	tags=36%, list=8%, signal=39%
CAF_YAPTAZ_ALL_UP	1087	0.39539856	2.0733216	0	0.00100357	tags=40%, list=24%, signal=49%
DASATINIB_DN	208	0.41236395	1.9052991	0	0.00597356	tags=39%, list=25%, signal=52%
UP_IN_STIFF_MATRIX_AND_PROLIF	40	0.5269615	1.8663244	0	0.00904752	tags=58%, list=32%, signal=84%
BERENJENO_ROCK_SIGNALING_NOT_VIA_RHOA_UP	29	0.5718424	1.8431051	0.00308642	0.01046525	tags=34%, list=11%, signal=39%
INTERFERON_IN_COCULTURE	21	0.59700376	1.8194805	0	0.0128522	tags=57%, list=10%, signal=63%
DASATINIB_NLF_VS_LCAF_DN	59	0.46670222	1.7978599	0	0.01549632	tags=49%, list=25%, signal=65%
CHANG_CYCLING_GENES	45	0.4761665	1.7479564	0.00342466	0.02394448	tags=53%, list=32%, signal=79%
CHEMOKINES	46	0.4477555	1.6653389	0.0131579	0.04840102	tags=24%, list=8%, signal=26%
SRC_GENES	40	0.46124756	1.6430713	0.01269841	0.05564842	tags=23%, list=12%, signal=26%
BERENJENO_TRANSFORMED_BY_RHOA_UP	516	0.32271415	1.6378736	0	0.05520763	tags=43%, list=30%, signal=59%
OVEREXPRESSED_G3_TO_G1	60	0.41245773	1.5733459	0.0203252	0.08515406	tags=35%, list=24%, signal=46%
BIOCARTA_WNT_PATHWAY	24	0.49468163	1.5402899	0.02686567	0.10252612	tags=25%, list=14%, signal=29%
DASATINIB_AND_QUIESCENCE_DN_	13	0.5902231	1.5396316	0.05865922	0.0985544	tags=62%, list=28%, signal=86%
IL5_UP	120	0.34324872	1.479517	0	0.13877364	tags=31%, list=20%, signal=38%
UP_IN_CAFS_FROM_COLON_CANCER_VS_OTHER_CELLS	189	0.31326473	1.4692802	0	0.1438278	tags=22%, list=15%, signal=26%
UP_IN_TUMOUR_VASCULATURE	22	0.47055393	1.4622253	0.0497076	0.14508827	tags=45%, list=20%, signal=57%
WILLERT_WNT_SIGNALING	19	0.4887554	1.4513843	0.0480226	0.14925931	tags=21%, list=5%, signal=22%
TPX2_METAGENE_VALIDATION_MSIGDB_CELLCYCLE	60	0.36732814	1.437243	0.02631579	0.15695032	tags=50%, list=32%, signal=73%
SANZ_ROCK_RESPONSIVE_GENES_TOP_600	159	0.31809756	1.4180127	0.00606061	0.16263932	tags=24%, list=14%, signal=28%
EGFR1_UP	86	0.34374973	1.395742	0.03167421	0.17869161	tags=27%, list=15%, signal=31%
SANZ_ROCK_RESPONSIVE_GENES_TOP_200	118	0.316888	1.382767	0.02051282	0.1856779	tags=20%, list=13%, signal=23%
B_CATENIN_GENES	42	0.38845336	1.3727274	0.07692308	0.19088629	tags=33%, list=21%, signal=42%
UP_IN_OVARIAN_CANCER_STROMA	181	0.2949167	1.3692465	0	0.18863927	tags=28%, list=22%, signal=36%
SECRETED_IN_PTEN_NULL_ETS2_TARGETS	17	0.48181775	1.3652046	0.1160221	0.18745367	tags=12%, list=6%, signal=13%
SECRETED_IN_PTEN_NULL_MIR_CONTROL_ONLY	30	0.4039811	1.3317623	0.09567902	0.2186328	tags=13%, list=7%, signal=14%
DASATINIB_CSR_DN	20	0.44028944	1.3092022	0.10447761	0.24180101	tags=50%, list=26%, signal=67%
WNT_SIGNATURE	58	0.33531976	1.2885729	0.07539683	0.2614431	tags=22%, list=9%, signal=24%
TSAO_FAK_RESPONSIVE_GENES_TOP_100	75	0.31272438	1.2758709	0.04705882	0.27267656	tags=29%, list=17%, signal=35%
WNT3A_DN_HLF	9	0.5346626	1.2734178	0.17985612	0.26954615	tags=33%, list=13%, signal=38%
ESR1_METAGENE_VALIDATION_MSIGDB_FRASOR_ER_UP	28	0.38853478	1.2609129	0.1574074	0.28064287	tags=21%, list=6%, signal=23%
IL1_UP	74	0.31007522	1.2485667	0.07287449	0.29375798	tags=23%, list=8%, signal=25%
WNT3A_2_DN	27	0.3811855	1.2483094	0.1411043	0.28734648	tags=26%, list=10%, signal=29%
CHANG_CORE_SERUM_RESPONSE_UP	61	0.31913802	1.2321556	0.11857708	0.3028827	tags=49%, list=33%, signal=73%
TSAO_FAK_RESPONSIVE_GENES_TOP_200	143	0.2776171	1.2282292	0.06060606	0.30267477	tags=32%, list=19%, signal=39%
EMT_SIGNATURE	59	0.3225245	1.2093763	0.16544117	0.32592374	tags=34%, list=20%, signal=42%
FINAK_BREAST_CANCER_SDPP_SIGNATURE	22	0.37436864	1.1880885	0.1934605	0.35692298	tags=23%, list=6%, signal=24%
RAS_GENES	164	0.25897092	1.1806686	0.05517241	0.36246186	tags=16%, list=12%, signal=18%
BECK_2008_DTF_SHORT	60	0.30819032	1.1794986	0.17037037	0.3571626	tags=27%, list=21%, signal=34%
SANZ_ROCK_RESPONSIVE_GENES_TOP_100	81	0.28906658	1.1793437	0.14893617	0.35046917	tags=20%, list=13%, signal=23%
REACTOME_SIGNALING_BY_WNT	56	0.31364837	1.1728323	0.16731517	0.3551215	tags=52%, list=40%, signal=86%
TNFA_NFKB_UP	109	0.27112076	1.1702441	0.12834224	0.35295495	tags=29%, list=15%, signal=34%
BECK_2008_DTF_LONG	153	0.2581587	1.1609442	0.09202454	0.3629104	tags=16%, list=11%, signal=18%
WNT_DN	9	0.49414393	1.1510755	0.3073048	0.37384212	tags=56%, list=14%, signal=65%
DTFD	31	0.3373959	1.14089	0.25787964	0.38507834	tags=26%, list=13%, signal=30%
UP_IN_NORMAL_VASCULATURE	42	0.30997026	1.1207839	0.22556391	0.4173743	tags=14%, list=10%, signal=16%
UP_IN_CAF	161	0.24336624	1.114415	0.14634146	0.4233718	tags=37%, list=25%, signal=48%
HYPOXIA2_UP	13	0.41652825	1.0884635	0.34770888	0.47211733	tags=38%, list=16%, signal=46%
KEGG_WNT_SIGNALING_PATHWAY	143	0.2419189	1.087065	0.25748503	0.467269	tags=20%, list=14%, signal=23%
IL2_UP	432	0.21722798	1.0866679	0.11666667	0.46057093	tags=20%, list=15%, signal=22%
WNT_GENES	18	0.36082	1.0485356	0.38055557	0.5409049	tags=17%, list=3%, signal=17%
UP_IN_COLON_CANCER_STROMA	185	0.22737516	1.046461	0.27083334	0.537197	tags=16%, list=11%, signal=17%
E2F3_GENES	140	0.2366722	1.0458014	0.3105263	0.5303604	tags=21%, list=16%, signal=25%
BASAKI_YBX1_TARGETS_UP	257	0.21614937	1.0435109	0.28723404	0.52801985	tags=30%, list=27%, signal=41%
TGFB_TARGET_GENES	75	0.25324655	1.0200565	0.425	0.5786391	tags=12%, list=8%, signal=13%
IL9_UP	25	0.32966632	1.017685	0.44207317	0.5757127	tags=16%, list=3%, signal=16%
SRFMALB	25	0.31936678	1.012284	0.42944786	0.57971025	tags=24%, list=20%, signal=30%
SFT	101	0.23679604	0.99852526	0.45454547	0.60697395	tags=37%, list=22%, signal=47%
STROMAL_SIGNATURE_RESISTANCE_TO_CHEMOTHERAPY	44	0.27804467	0.9945592	0.4602649	0.6083814	tags=16%, list=11%, signal=18%
CD83_METAGENE_VALIDATION_MSIGDB_CD40_PATHWAYS	12	0.36996454	0.97242063	0.45576409	0.6577655	tags=25%, list=14%, signal=29%
YAP	60	0.24911	0.96994513	0.50763357	0.65552783	tags=25%, list=14%, signal=29%
BERENJENO_TRANSFORMED_BY_RHOA_FOREVER_DN	31	0.28467947	0.95439863	0.5379939	0.68731874	tags=42%, list=23%, signal=54%
SECRETOME_SIGNATURE	37	0.2717667	0.95388365	0.50859106	0.67934996	tags=11%, list=7%, signal=12%
GZMA_METAGENE_VALIDATION_MSIGDB_TCRA_PATHWAY	9	0.39855123	0.945988	0.55949366	0.69006723	tags=44%, list=13%, signal=51%
STAT3_EXPRESSION_SIGNATURE	10	0.3754142	0.9113359	0.6065163	0.76580864	tags=20%, list=5%, signal=21%
FABP4_METAGENE_VALIDATION_MSIGDB_FATTYACID_DEGRADATI ON	22	0.29789293	0.90943056	0.5862069	0.76000625	tags=36%, list=22%, signal=47%
HYPOXIA2_DN	6	0.42478168	0.90356815	0.5704057	0.7633641	tags=67%, list=18%, signal=81%
BREAST_STROMA	9	0.38067257	0.9009057	0.60097325	0.75971454	tags=11%, list=0%, signal=11%
WNT3A_UP_HLF	60	0.23006903	0.8817408	0.6967509	0.7913671	tags=13%, list=6%, signal=14%
BERENJENO_TRANSFORMED_BY_RHOA_FOREVER_UP	18	0.3065489	0.88030595	0.6426593	0.7842404	tags=22%, list=13%, signal=26%
YAP_TAZ_COMMON	544	0.16726863	0.8379164	1	0.8580315	tags=24%, list=21%, signal=29%
BREAST_EPITHELIUM	9	0.35247335	0.83541495	0.6580189	0.8518681	tags=22%, list=6%, signal=24%
IGS_INVASIVENESS_GENE_SIGNATURE	113	0.18892017	0.8256004	0.8995215	0.8581976	tags=39%, list=28%, signal=54%
REACTOME_NOTCH_HLH_TRANSCRIPTION_PATHWAY	13	0.29949048	0.7658761	0.7622549	0.9369401	tags=23%, list=15%, signal=27%
IL5_DN	8	0.3259288	0.7588544	0.77057356	0.9337192	tags=25%, list=12%, signal=28%
IGF1	252	0.15659869	0.7533783	1	0.9299305	tags=23%, list=25%, signal=30%
MX_METAGENE_VALIDATION_MSIGDBIFNPATHWAY	8	0.3296809	0.7530671	0.7762238	0.9194759	tags=13%, list=5%, signal=13%
NGUYEN_NOTCH1_TARGETS_UP	21	0.2351851	0.71200347	0.8785311	0.9475462	tags=33%, list=26%, signal=45%
SECRETED_IN_PTEN_NULL_MIR320_AND_CONTROL	53	0.17200081	0.6451299	0.99298245	0.97928137	tags=19%, list=19%, signal=23%
SECRETOME_SIGNATURE_ETS2_TARGETS	13	0.18794869	0.5035284	0.98039216	1	tags=15%, list=18%, signal=19%
SECRETED_IN_PTEN_NULL_MIR320_ONLY	16	0.17385834	0.49005717	0.9895013	0.9957234	tags=38%, list=32%, signal=55%
TAZ_INDUCED_GENES	1462	0.19637047			1	tags=30%, list=26%, signal=37%

Ferrari *et al*. Supplementary Information. Supplementary Table 1 (continued).

NAME	SIZE	ES	NES	NOM p-val	FDR q-val	LEADING EDGE
CAF_BCAT_ALL_DN	203	-0.6452891	-2.4904265	0	0	tags=61%, list=14%, signal=70%
DASATINIB_CSR_UP	16	-0.6712642	-1.7059835	0.00802568	0.01617807	tags=56%, list=23%, signal=73%
HGF_DN	18	-0.6019741	-1.6121591	0.01926164	0.04520309	tags=44%, list=14%, signal=52%
DASATINIB_UP	141	-0.4199701	-1.5733957	0.00120337	0.06164124	tags=39%, list=22%, signal=49%
CAF_YAPTAZ_ALL_DN	879	-0.3575035	-1.4962401	0	0.11768349	tags=33%, list=19%, signal=39%
DN_IN_STROMA_WHEN_METS	9	-0.6116958	-1.3194784	0.12798634	0.43185088	tags=33%, list=10%, signal=37%
SRFMALA	120	-0.3623683	-1.3185855	0.05882353	0.40576732	tags=29%, list=23%, signal=38%
L1601P_GENES_UP_NOTCH_UP	123	-0.3507254	-1.2888318	0.06976745	0.46668872	tags=28%, list=14%, signal=33%
CSF1R_CORE_SHORT	97	-0.3534102	-1.2784195	0.07850708	0.47026992	tags=35%, list=20%, signal=44%
WNT_UP	33	-0.4312946	-1.2775955	0.15827338	0.42270362	tags=36%, list=16%, signal=43%
TGFB	182	-0.3267436	-1.2525476	0.07339449	0.47281578	tags=26%, list=20%, signal=33%
UP_IN_STROMA_WHEN_METS	38	-0.4049588	-1.2514036	0.15417255	0.45366746	tags=21%, list=11%, signal=24%
CLCA2_METAGENE_VALIDATION_MSIGDB_NELSON_ANDROGEN_UP	53	-0.3850158	-1.2508042	0.16576087	0.434756	tags=45%, list=30%, signal=65%
CSF1R_CORE_LONG	182	-0.3243769	-1.2391858	0.09132948	0.4458404	tags=31%, list=20%, signal=38%
IL4_UP	166	-0.3151875	-1.206133	0.11729858	0.5251247	tags=25%, list=16%, signal=29%
NICD_GENES_UP_NOTCH_UP	675	-0.2810855	-1.1778785	0.04786151	0.59500396	tags=21%, list=16%, signal=24%
HYPOXIA	48	-0.3670543	-1.1721606	0.23021583	0.59306717	tags=27%, list=20%, signal=34%
UP_IN_NF	109	-0.3112574	-1.1292586	0.23321123	0.725402	tags=34%, list=24%, signal=44%
IL6_UP	13	-0.462529	-1.1264745	0.31061807	0.7096828	tags=38%, list=23%, signal=50%
DASATINIB_AND_QUIESCENCE_UP	19	-0.4189483	-1.1171187	0.3114504	0.72052497	tags=32%, list=18%, signal=38%
TEAD_DEPENDENT_YAP_TARGET_GENES_COMMON_BETWEEN_YAP_AND_TAZ	31	-0.379337	-1.1097721	0.32900432	0.7234895	tags=42%, list=28%, signal=58%
YAP_TAZ_CONSERVED	56	-0.3360014	-1.1042212	0.29781422	0.72082245	tags=25%, list=20%, signal=31%
NOTCH_UP	17	-0.4314299	-1.0987349	0.3423138	0.7188801	tags=18%, list=4%, signal=18%
MAMMOSPHERE	24	-0.3941557	-1.075229	0.3897059	0.78576946	tags=33%, list=19%, signal=41%
UP_IN_BREAST_CANCER_STROMA_FINAK	167	-0.2836145	-1.0698419	0.322807	0.7833638	tags=22%, list=13%, signal=25%
BERENJENO_TRANSFORMED_BY_RHOA_DN	382	-0.2570463	-1.0536366	0.34632036	0.82469773	tags=37%, list=30%, signal=51%
TGFB_PADUA	130	-0.2792285	-1.0374708	0.37875	0.8667753	tags=25%, list=25%, signal=34%
UP_IN_BREAST_CANCER_STROMA_KARNOUB	155	-0.2685953	-1.0280514	0.41970804	0.8799523	tags=30%, list=23%, signal=38%
CHANG_CORE_SERUM_RESPONSE_DN	27	-0.3593718	-1.0273616	0.41420117	0.8592859	tags=41%, list=28%, signal=57%
UP_IN_YAP_LIVER	709	-0.2465016	-1.0260534	0.41598362	0.84172153	tags=20%, list=20%, signal=24%
BERENJENO_TRANSFORMED_BY_RHOA_REVERSIBLY_UP	8	-0.4867267	-1.0144619	0.47440273	0.86324346	tags=25%, list=9%, signal=27%
UP_IN_PYMT_CAFS_VS_NF	190	-0.2611817	-1.0133586	0.44	0.846394	tags=19%, list=17%, signal=23%
WNT3A_2_UP	30	-0.3496065	-1.0106475	0.45760235	0.8358374	tags=37%, list=26%, signal=50%
HGF_UP	56	-0.3084348	-1.0050954	0.445215	0.8350834	tags=14%, list=9%, signal=16%
TEAD_DEPENDENT_YAP_TARGET_GENES_UNIQUE_TO_YAP	21	-0.3665148	-0.9982209	0.46270928	0.84094113	tags=29%, list=23%, signal=37%
DOWN_IN_STIFF_MATRIX	52	-0.3020471	-0.978011	0.4979592	0.8911025	tags=27%, list=22%, signal=35%
YAPTAZ	66	-0.2920919	-0.9749934	0.5045632	0.8817606	tags=21%, list=20%, signal=26%
TSAO_FAK_RESPONSIVE_GENES__TOP_600	385	-0.2404036	-0.9706699	0.5729387	0.8767035	tags=16%, list=12%, signal=18%
DASATINIB_NLF_VS_LCAF_UP	26	-0.3404789	-0.968454	0.5263158	0.8656578	tags=42%, list=27%, signal=58%
ADM_METAGENE_VALIDATION_MSIGDB_HIFPATHWAY	12	-0.4106454	-0.9651203	0.516184	0.8585951	tags=8%, list=1%, signal=8%
NFKB	159	-0.2536896	-0.9641835	0.5400239	0.84455043	tags=25%, list=19%, signal=30%
VANTVEER_BREAST_CANCER_POOR_PROGNOSIS	41	-0.3101444	-0.9641796	0.5227606	0.82799065	tags=12%, list=6%, signal=13%
NOTCH_SIGNALING_PATHWAY	12	-0.4064188	-0.9637584	0.5152	0.8134396	tags=67%, list=37%, signal=105%
PDGF	13	-0.3970734	-0.959429	0.5223421	0.8110043	tags=46%, list=35%, signal=71%
NICD_GENES_DOWN_NOTCH_DOWN	841	-0.2270055	-0.9567062	0.64658636	0.80407107	tags=21%, list=19%, signal=25%
DCN_METAGENE_VALIDATION_MSIGDB_TGFB_SIGNALLING_PATHWAY	47	-0.2995402	-0.9546415	0.5396384	0.7949489	tags=34%, list=26%, signal=46%
WNT_TARGET_GENES	92	-0.2616469	-0.9226178	0.625323	0.8675414	tags=22%, list=15%, signal=25%
RANKL_UP	49	-0.2823421	-0.9074553	0.6181047	0.8911211	tags=18%, list=12%, signal=21%
BASAKI_YBX1_TARGETS_DN	313	-0.2196964	-0.8839042	0.7758433	0.9342578	tags=22%, list=20%, signal=28%
MYC_GENES	110	-0.2431145	-0.8727415	0.734414	0.94532514	tags=15%, list=15%, signal=18%
IL3_UP	16	-0.3386577	-0.8627421	0.65217394	0.95195925	tags=44%, list=31%, signal=63%
L1601P_GENES_DOWN_NOTCH_DOWN	37	-0.2743906	-0.8486379	0.6896552	0.96678096	tags=16%, list=8%, signal=18%
TGFB_ADORNO	136	-0.2258532	-0.8369581	0.8171913	0.9754914	tags=25%, list=21%, signal=31%
IL1_DN	33	-0.2794975	-0.8365638	0.7349927	0.9608314	tags=24%, list=16%, signal=29%
HOSHIDA_LIVER_CANCER_SUBCLASS_S1	220	-0.2094288	-0.8198768	0.87093157	0.9768792	tags=15%, list=16%, signal=18%
REACTOME_SIGNALING_BY_NOTCH	16	-0.3188872	-0.8068203	0.7464342	0.98555046	tags=19%, list=14%, signal=22%
IL2_DN	243	-0.2042531	-0.8064457	0.9065315	0.9712625	tags=14%, list=15%, signal=17%
YAP_INDUCED_GENES	997	-0.1900561	-0.7990856	0.9899295	0.96904176	tags=21%, list=25%, signal=26%
IL4_DN	71	-0.2337412	-0.7967912	0.8425197	0.95845425	tags=25%, list=20%, signal=32%
RANKL_DN	15	-0.3096829	-0.7855121	0.7832512	0.9613825	tags=27%, list=16%, signal=32%
YAP_UP	976	-0.1844274	-0.7763023	0.9949495	0.96031094	tags=20%, list=25%, signal=26%
EGFR1_DN	42	-0.2505257	-0.7663638	0.84791964	0.9591901	tags=33%, list=30%, signal=48%
NOTCH_DN	5	-0.4084545	-0.7524144	0.7775832	0.96273273	tags=40%, list=14%, signal=47%
BERENJENO_TRANSFORMED_BY_RHOA_REVERSIBLY_DN	28	-0.2429434	-0.7022601	0.89301634	0.997088	tags=14%, list=20%, signal=18%
TNFA_NFKB_DN	7	-0.3372279	-0.6904588	0.85045046	0.9918127	tags=14%, list=3%, signal=15%
IL7_UP	20	-0.2539844	-0.6809104	0.8844444	0.98512787	tags=30%, list=24%, signal=40%
BERENJENO_ROCK_SIGNALING_NOT_VIA_RHOA_DN	48	-0.2118798	-0.6757336	0.941094	0.9754793	tags=29%, list=28%, signal=40%
UP_IN_STIFF_MATRIX	42	-0.2114296	-0.6589775	0.9431818	0.97163904	tags=24%, list=28%, signal=33%
NGUYEN_NOTCH1_TARGETS_DN	63	-0.1935534	-0.6520087	0.97705805	0.9626167	tags=29%, list=29%, signal=40%
KEGG_NOTCH_SIGNALING_PATHWAY	46	-0.1897905	-0.6022859	0.97910863	0.96780443	tags=46%, list=40%, signal=76%

Supplementary Table 1. GSEA report. Table describing the GSEA analysis including gene set name, size, enrichment score (ES), normalised enrichment score (NES), p value, False discovery rate (FDR) q value, and leading edge analysis.

Ferrari *et al*. Supplementary Information. Supplementary Table 2.

Antibody	Company	Cat. No	Clonal	Dilution					
				IF	WB	IHC	IP	FACS	Tissue IF
Alexa Fluor® 488 Donkey Anti-Mouse IgG (H+L) Antibody	invitrogen	A21202	polyclonal	1:300					
Alexa Fluor® 488 Goat Anti-Rabbit IgG (H+L) Antibody	invitrogen	A11008	polyclonal	1:300					
Alexa Fluor® 555 Donkey Anti-Mouse IgG (H+L)	invitrogen	A31570	polyclonal	1:300					
Alexa Fluor® 555 Goat Anti-Rabbit IgG (H+L) Antibody	invitrogen	A21428	polyclonal	1:300					
CD140a (PDGFRa) Antibody - PE anti-mouse	BioLegend	135905	polyclonal					1:100	
CD31 Clone MEC 13.3 (RUO), 25ug - APC Rat Anti-Mouse	BD Pharmingen™	561814	monoclonal					1:200	
CD326 (Ep-CAM) Antibody, 25ug - PE/Cy7 anti-mouse	BioLegend	118215	monoclonal					1:100	
CD45 Clone 30-F11 (RUO), 0.1mg - FITC Rat Anti-Mouse	BD Pharmingen™	553079	monoclonal					1:200	
DAPI			n/a	1:500					
Dkk1	Proteintech	21112-1-AP	polyclonal						1:200
DKK2 antibody - aminoterminal end	Abcam	ab38594	polyclonal		1:1000				
DKK3 (human)	Sigma/Atlas	HPA011868	polyclonal			1:100			
DKK3 (human)	R&D	AF1118	polyclonal		1:1000				
Dkk3 (mouse) Affinity Purified Polyclonal Ab, 100ug	R&D	AF948	polyclonal		1:1000				
DKK-3 H-130	Santa Cruz	sc-25518	polyclonal			1:100			
Fibroblast activation protein, alpha antibody	Abcam	ab28244	polyclonal	1:100	1:1000				
Fibronectin	Dako	A0245	polyclonal			1:2000			
FLAG M2	Cell Signalling	14793	monoclonal				1:50		
FLAG M2	Sigma	F1804	monoclonal		1:1000				
GAPDH (14C10) Rabbit mAB	cell signaling	3683	monoclonal		1:2000				
HSF1	Cell Signalling	4356S	polyclonal		1:1000				
HSF1 Ab-1 (Clone 4B4)	Thermo Scientific	RT-405-P1	polyclonal	1:100	1:1000				
Kremen-1 (human) Antibody, 25ug	R&D	MAB2127-SP	monoclonal		1:1000		1:50		
Kremen-1 (mouse) Antibody	R&D	AF1647-SP	polyclonal		1:1000				
Laminin	Dako	S0809	polyclonal			1:100			
LRP6 (C47E12) Rabbit mAb	Cell Signalling	#3395	monoclonal		1:1000				
Non-phospho (Active) β-Catenin (Ser33/37/Thr41)	Cell Signaling	#8814	monoclonal	1:100	1:1000	1:100			
Phalloidin-FITC	Sigma	P1951	n/a	1:500					
Phalloidin-TRITC	Sigma	P1951	n/a	1:500					
phospho-Myosin Light Chain 2 (Thr18/Ser19)	cell signaling	3674	polyclonal		1:1000				
Phospho-Src Family Kinases (Y416)	Cell Signaling	#2101	polyclonal		1:1000				
phospho-YAP (ser127)	Cell Signaling	#4911	polyclonal		1:1000				
Polyclonal Goat anti-Mouse immunoglobulins HRP	DAKO	P044701-2	polyclonal		1:5000				
Polyclonal Goat anti-Rabbit immunoglobulins HRP	DAKO	P044801-2	polyclonal		1:5000				
Polyclonal Rabbit anti-Goat Immunoglobulins HRP	Dako	P0449	polyclonal		1:5000				
S100A4 (FSP1)	Abcam	ab27957	polyclonal	1:100	1:1000				1:200
ß-catenin (E-5)	Santa Cruz	sc-7963	monoclonal	1:100	1:1000	1:100			1:200
TAZ (V386)	Cell Signaling	#4883	polyclonal	1:100	1:1000				
YAP	Santa cruz	sc-101199	monoclonal	1:200	1:2000				1:200
αSMA	Sigma	A2547	monoclonal	1:500	1:5000				1:200
αSMA	Dako	M0851	monoclonal			1:200			
β-Tubulin I	Sigma	T7816	monoclonal		1:5000				

Supplementary Table 2. Antibodies. Name, company, catalogue number and working dilutions of all the antibodies used in the study.

Ferrari *et al*. Supplementary Information. Supplementary Table 3.

NAME	target	sequence
sh HuDKK3_1-TRCN0000033398	human shDKK3	CCGGGACACGAAGGTTGGAAATAATCTCGAGATTATTTCCAACCTTCGTGTCTTTTTG
sh HuDKK3_2-TRCN0000033396	human shDKK3	CCGGCCCAGCATGTACTGCCAGTTTCTCGAGAAACTGGCAGTACATGCTGGGTTTTTG
sh HuDKK3_3-TRCN0000033395	human shDKK3	CCGGGCAAACTTACCTCCCAGCTATCTCGAGATAGCTGGGAGGTAAGTTTGCTTTTTG
sh HuDKK3_4-TRCN0000033394	human shDKK3	CCGGGCACCGAGAAATTCACAAGATCTCGAGATCTTGTGAATTTCTCGGTGCTTTTTG

Supplementary Table 3. shRNAs. Gene, name and sequences of the single shRNAs used in the study.

Ferrari *et al*. Supplementary Information. Supplementary Table 4.

REF	NAME	Individual siRNA	Sequence
MU-060631-00-0002	siGENOME Mouse DKK3	D-060631-01, Dkk3	UCAAUGAGAUGUUUCGAGA
		D-060631-02, Dkk3	AAGCUUACCUCCCAACUAU
		D-060631-03, Dkk3	AGACCAGGGUGGGAAAUAA
		D-060631-04, Dkk3	GAGGAGCCAUGAAUGUAUC
MU-018352-01-0005	siGENOME Human DKK3	D-018352-01, Dkk3	AAACUUACCUCCCAGCUAU
		D-018352-02, Dkk3	CCGAGAAAUUCACAAGAUA
		D-018352-03, Dkk3	GGACACGCAGCACAAAUUG
		D-018352-04, Dkk3	UCAAUGAGAUGUUCCGCGA
MU-060757-01-005	siGENOME Mouse DKK2	D-060757-01, Dkk2	GCAAACCAGUGCUCCAUCA
		D-060757-02, Dkk2	CAACCGAUCUGCAGGCAUG
		D-060757-03, Dkk2	GACCUGGGAUGGCAGAAUC
		D-060757-04, Dkk2	CCUGGUACCGCUGCAAUA
M-003846-01-0005	siGENOME Human Kremen1	D-003846-01, Kremen1	GAGCACAACUAUUGCAGAA
		D-003846-02, Kremen1	GAACGAGACUUUCCAGCAU
		D-003846-03, Kremen1	UCACAGCCAUUGUAGCAAA
		D-003846-04, Kremen1	CAACGUCUCUCUGGACUUC
M-046771-01-0005	siGENOME Mouse Kremen1 (SMART pool)	D-046771-01, Kremen1	AGUCAGAGAUUCAAGUUUG
		D-046771-02, Kremen1	UCACAGCAGUUGUCGCAAA
		D-046771-03, Kremen1	GGAACAAUCCUGACUACUG
		D-046771-04, Kremen1	CCAGGGAUUGCUGUGUUG
M-049736-00-0005	siGENOME Mouse Kremen2 (SMARTpool)	D-049736-01, Kremen2	GCGCAUAACUUCUGUAGGA
		D-049736-02, Kremen2	GAACGGCGCUGACUACCGA
		D-049736-03, Kremen2	GCACAGGCUUCGAUAGGUG
		D-049736-04, Kremen2	GCUGGACGCCCUUGUCUUU
MU-041057-01-0002	siGENOME Mouse Wwtr1	D-041057-01, Wwtr1	GGCCAGAGAUACUUCCUUA
		D-041057-02, Wwtr1	CCACAGGGCUCAUGAGUGU
		D-041057-03, Wwtr1	GGAUUAGGAUGCGUCAAGA
		D-041057-04, Wwtr1	CGAGAUGGAUACAGGUGAA
MU-046247-01-0002	siGENOME Mouse Yap1	D-046247-01, Yap1	GGAGAAGUUUACUACAUAA
		D-046247-02, Yap1	CCACCAAGCUAGAUAAAGA
		D-046247-03, Yap1	GAGAUGCAAUGAACAUAGA
		D-046247-04, Yap1	CAAUAGUUCCGAUCCCUUU
M-040650-01-005	siGENOME Mouse LRP5	D-040650-01, Lrp5	GCACAAAGGCCACACUAUA
		D-040650-02, Lrp5	GGACUGACCUGGACACCAA
		D-040650-03, Lrp5	UCAAAGCCAUCAACUAUGA
		D-040650-04, Lrp5	CCAACGACCUCACCAUUGA
M-040651-01-0005	siGENOME Mouse LRP6	D-040651-01, LRP6	GGACAGACCUGGACACUAA
		D-040651-02, LRP6	GGAAAGACCUGCAAAGAUG
		D-040651-03, LRP6	UCACAUAUCUGCCUUGUAA
		D-040651-04, LRP6	GGACGGAUCUGACCGAGUA
M-040628-00-0005	siGENOME Mouse Ctnnb1	D-040628-01, Ctnnb1	GAUCUUAGCUUAUGGCAAU
		D-040628-02, Ctnnb1	GCAAGUAGCUGAUAUUGAC
		D-040628-03, Ctnnb1	CAGUGGCCUGGUUUGAUA
		D-040628-04, Ctnnb1	GAACGCAGCAGCAGUUUGU
M-040660-01-0005	siGENOME Mouse Hsf1	D-M-040660-01, Hsf1	GCUAAGUGAUCACCUGGAU
		D-M-040660-02, Hsf1	CAAGUAUGGUCGACAGUAC
		D-M-040660-02, Hsf1	AGAACGAGCUAAGUGAUCA
		D-M-040660-02, Hsf1	UGCGGCAGCUCAACAUGUA
M-063467-01-0005	siGENOME Mouse Lats1	D-063467-01, Lats1	GCAGAGUACUAGCAAAUUU
		D-063467-02, Lats1	GCAGCUGCCAGGCCUAUUA
		D-063467-03, Lats1	GGAACAGUCAUAACAUGGA
		D-063467-04, Lats1	GAAACGUUCCUCAGUCGAU
M-044602-01-0005	siGENOME Mouse Lats2	D-044602-01, Lats2	GCGGCAAUAUUUAGACUUU
		D-044602-02, Lats2	GAAAUAGCCGGCAGCGAUU
		D-044602-03, Lats2	GGGCCAAGACGGACAAGUC
		D-044602-04, Lats2	UCAGGGAAAUCCGAUAUUC

Supplementary Table 4. siRNas. Name, catalogue number, and sequence of the single siRNAs used in the study.

qPCR Primers	
**Primer name**	**Sequence**
hRPLP1_F	AGCCTCATCTGCAATGTAGGG
hRPLP1_R	TCAGACTCCTCGGATTCTTCTTT
mRplp1-F	ACCGTGCCGGCAGTCTACAG
mRplp1-r	ATGTTGACATTGGCCAGAGCCTTG
hGAPDH_F	GGCAAATTCCATGGCACCG
hGAPDH_R	GCATCGCCCCACTTGATTTT
mGapdh_F	GTGCAGTGCCAGCCTCGTCC
mGapdh_R	GCCACTGCAAATGGCAGCCC
mDkk3-TRC_F	CTCGGGGGTATTTTGCTGTGT
mDkk3-TRC_R	TCCTCCTGAGGGTAGTTGAGA
hDKK3a_F	AGGACACGCAGCACAAATTG
hDKK3a_R	CCAGTCTGGTTGTTGGTTATCTT
mHsf1_F4	AACGTCCCGGCCTTCCTAA
mHsf1_R4	AGATGAGCGCGTCTGTGTC
mHsf1_F1	TTGACTCCATCCTTCGAGAGAG
mHsf1_R1	GTCAGGCAGGCTCATGTCG
mKrt18_F	TCAAGATCATCGAAGACCTGAGG
mKrt18_R	GCGCATGGCTAGTTCTGTC
mPdgfra_F	AGAGTTACACGTTTGAGCTGTC
mPdgfra_R	GTCCCTCCACGGTACTCCT
mPtprc_F	ACGCTGGTGCTCTATGCAAG
mPtprc_R	TCAGTTGCTGCCCATTCATCA
mPecam1_F	GTTTTCGCTACATGACTGCACA
mPecam1_R	AGGTTGTCCAACTGACATCTTTC
mFap_F	GTCACCTGATCGGCAATTTGT
mFap_R	CCCCATTCTGAAGGTCGTAGAT
mFn1_f	GCTCAGCAAATCGTGCAGC
mFn1_r	CTAGGTAGGTCCGTTCCCACT
mDkk3-promoter_F	AGGATGGACACCAACAGTCC
mDkk3-promoter_R	TCAGGTACACAGCCCATTTTC
mDkk3-Enhancer_F	CCAGGGTTTGTTCTCAAGGA
mDkk3-Enhancer_R	ACTGTGTGGGCCTAGAATGG
mRilpl_F	AGAACCTTCTGGAAGCACGA
mRilpl_R	GCTTCTGGCAACAAGAGGAG

Supplementary Table 5. Primers. Names and sequences (forward and reverse) of the paired oligos used in this study for qRT-PCR. The name contains the target gene; h stands for human, m for murine; F for forward and R for Reverse.

Ferrari *et al*. Supplementary Information. Supplementary Table 6.

Term	Overlap	P-value	Adjusted P-value	Old P-value	Old Adjusted P-value	Z-score	Combined Score	Genes
pou4f1_20376082_fetal_liver_lof_mouse_gpl1261_gds4042_down	1/144	0.0072	0.038	0.004257	0.022424	-2.61051	12.87939	DKK3
nrf1_22586274_liver_lof_mouse_gpl4134_gse35124_down	1/109	0.00545	0.038	0.003229	0.022424	-2.21245	11.53159	DKK3
hnf1b_16297991_hek293_embryonic_gof_mouse_gpl96_gds1499_up	1/170	0.0085	0.038	0.00502	0.022424	-1.9209	9.158274	DKK3
creb1_22108299_lung_lof_mouse_gpl1261_gds3660_up	1/200	0.01	0.038	0.005901	0.022424	-1.77125	8.156887	DKK3
tcof1_15522210_neuroblastoma_lof_mouse_gpl339_gds998_down	1/183	0.00915	0.038	0.005402	0.022424	-1.72895	8.115714	DKK3
gata4_16914500_e9dot5_atrioventricular_canal_lof_mouse_gpl1261_gds3663_up	1/354	0.0177	0.05605	0.010422	0.033004	-1.61784	6.526694	DKK3
smarcc2_00000000_e12dot5_embryonic_cortex_lof_mouse_gpl6887_gse45629_up	1/879	0.04395	0.071031	0.025835	0.04175	-1.83663	5.738911	DKK3
pou5f1_20526341_human_embryonic_stem_cells_hesc_lof_human_gpl6947_gse21135_up	1/612	0.0306	0.071031	0.017997	0.04175	-1.30692	4.556911	DKK3
glis2_17618285_kidney_lof_mouse_gpl2897_gds2817_up	1/913	0.04565	0.071031	0.026833	0.04175	-1.40414	4.334235	DKK3
tcof1_15522210_neuroblastoma_gof_mouse_gpl339_gds998_down	1/742	0.0371	0.071031	0.021813	0.04175	-1.21492	4.002126	DKK3
zfpm2_19411579_heart_lof_mouse_gpl1261_gds3659_up	1/882	0.0441	0.071031	0.025923	0.04175	-1.28042	3.996579	DKK3
ccnd1_18413728_imr_neuroblastoma_lof_human_gpl570_gse8866_up	1/809	0.04045	0.071031	0.02378	0.04175	-1.21973	3.912517	DKK3
creb1_22108299_heart_left_ventricle_lof_mouse_gpl1261_gds3660_up	1/972	0.0486	0.071031	0.028566	0.04175	-1.08551	3.282717	DKK3
hsf1_17216044_hela_lof_human_gpl571_gds1733_up	1/2136	0.1068	0.144943	0.062739	0.085145	-1.28888	2.882952	DKK3
bmi1_17452456_medulloblastoma_lof_human_gpl570_gds2724_up	1/7126	0.3563	0.396414	0.209236	0.232791	-1.24068	1.280359	DKK3
pcgf2_17452456_medulloblastoma_lof_human_gpl570_gds2724_up	1/7511	0.37555	0.396414	0.220539	0.232791	-1.26344	1.237366	DKK3
ets_00000000_2008_ovarian_cancer_cells_gof_human_gpl6244_gse21129_up	1/4717	0.23585	0.298743	0.138512	0.175449	-0.79622	1.150189	DKK3
rnf2_20805357_u2os_osteosarcoma_lof_human_gpl570_gse23035_up	1/5469	0.27345	0.324722	0.16059	0.1907	-0.83405	1.081454	DKK3
sin3a_22783022_mcf7_lof_human_gpl570_gds4388_up	1/4976	1	1	1	1	-1.11414	-9.5E-12	DKK3

Supplementary Table 6. **TF-LOF Expression from GEO dataset for DKK3 from Enrich**. Name, description and statistics of TFs that affect DKK3 expression after loss-of-function.
