# Supplementary material for: Dickkopf-3 links HSF1 and YAP/TAZ signalling to control aggressive behaviours in cancer-associated fibroblasts
Source: Nat Commun. 2019 Jan 10;10:130. doi: 10.1038/s41467-018-07987-0 (PMC6328607; doi:10.1038/s41467-018-07987-0)
Supplement: Supplementary file 3 — Description of Additional Supplementary Files [file 41467_2018_7987_MOESM3_ESM.docx]

**Description of Additional Supplementary Files**

**File Name**: Supplementary Data 1

**Description**: Transcription Factors that potentially bind the DKK3 Promoter, as obtained from ChEA 2016 dataset from Enrich (for DKK3). Information includes name of TF, description and statistics.

**File Name:** Supplementary Data 2.

**Description**: List of curated Gene sets used in this study. Information includes Name, Reference and Gene list for all gene sets.

**File Name**: Supplementary Movie 1.

**Description:** WT-CAFs generate environments that promote cancer cell and CAF motility in vivo. Representative time-lapse video generated from intravital imaging of a tumour growing subcutaneously in a living mouse: TS1 (red), CAF-WT (green), and collagen second harmonic (blue). Images were acquired every 15 min. Arrows indicate areas where either cancer cells of CAFs are moving. Scale bar, 50 μm.

**File Name**: Supplementary Movie 2

**Description:** DKK3-KO-CAFs are defective in promoting cancer cell and CAF motility in vivo. Representative time-lapse video generated from intravital imaging of a tumour growing subcutaneously in a living mouse: TS1 (red), CAF-KO.9 (green), and collagen second harmonic (blue). Images were acquired every 15 min. Scale bar, 50 μm.
